# Phase-Coherent Dynamics of Quantum Devices with Local Interactions

**DOI:** 10.3390/e22080847

**Published:** 2020-07-31

**Authors:** Michele Filippone, Arthur Marguerite, Karyn Le Hur, Gwendal Fève, Christophe Mora

**Affiliations:** 1 Department of Quantum Matter Physics, University of Geneva 24 Quai Ernest-Ansermet, CH-1211 Geneva, Switzerland; 2 Department of Condensed Matter Physics, Weizmann Institute of Science, Rehovot 7610001, Israel; arthur.marguerite@weizmann.ac.il; 3 CPHT, CNRS, Institut Polytechnique de Paris, Route de Saclay, 91128 Palaiseau, France; karyn.le-hur@polytechnique.edu; 4 Laboratoire de Physique de l’Ecole Normale Supérieure, ENS, Université PSL, CNRS, Sorbonne Université, Université de Paris, F-75005 Paris, France; gwendal.feve@lpa.ens.fr; 5 Laboratoire Matériaux et Phénomènes Quantiques, CNRS, Université de Paris, F-75013 Paris, France; christophe.mora@u-paris.fr

**Keywords:** dynamics of strongly correlated quantum systems, quantum transport, mesoscopic physics, quantum dots, quantum capacitor, local fermi liquids, kondo effect, coulomb blockade

## Abstract

This review illustrates how Local Fermi Liquid (LFL) theories describe the strongly correlated and coherent low-energy dynamics of quantum dot devices. This approach consists in an effective elastic scattering theory, accounting exactly for strong correlations. Here, we focus on the mesoscopic capacitor and recent experiments achieving a Coulomb-induced quantum state transfer. Extending to out-of-equilibrium regimes, aimed at triggered single electron emission, we illustrate how inelastic effects become crucial, requiring approaches beyond LFLs, shedding new light on past experimental data by showing clear interaction effects in the dynamics of mesoscopic capacitors.

## 1. Introduction

The manipulation of local electrostatic potentials and electron Coulomb interactions has been pivotal to control quantized charges in solid state devices. Coulomb blockade [1,2,3] has revealed to be a formidable tool to trapping and manipulating single electrons in localized regions behaving as highly tunable artificial impurities, so called quantum dots. Beyond a clear practical interest, which make quantum dots promising candidates to become the building block of a quantum processor [4,5,6], hybrid [7] quantum dot systems also became a formidable platform to address the dynamics of many-body systems in a controlled fashion, and a comprehensive theory, which could establish the role of Coulomb interactions when these systems are strongly driven out of equilibrium, is still under construction.

**Figure 1:**
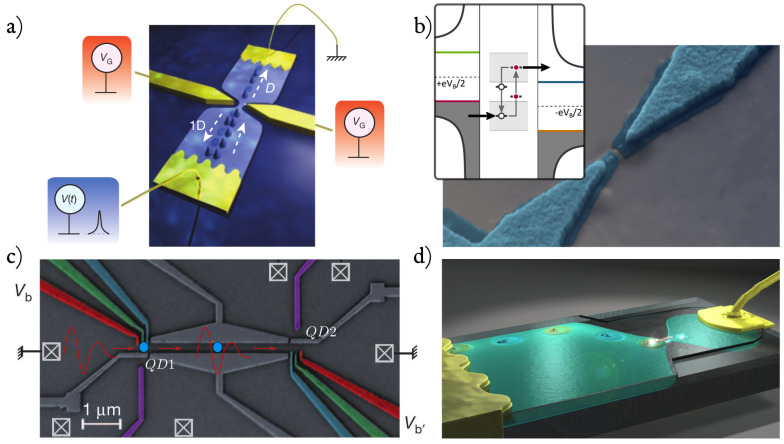
Some recent experiments achieving real-time control of single electrons. (**a**) Leviton generation by a Lorentzian voltage pulse in metallic contacts, generating a noiseless wave-packet carrying the electron charge *e* [8,9,10,11,12,13]. This wave-packet is partitioned on a Quantum Point Contact (QPC), whose transmission *D* is controlled by the split-gate voltage $V_{G}$. (**b**) Single quantum level electron turnstile [18,19]. Two superconductors, biased by a voltage $V_{B}$, are connected by a single-level quantum dot. Inset—Working principle of the device: A gate voltage controls the orbital energy of the quantum dot, which is filled by the left superconductor and emptied in the right one. (**c**) Long-range single-electron transfer via a radio-frequency pulse between two distant quantum dots QD1 and QD2 [14,15,16,17]. The electron “surfs” along the moving potential generated by the radio-frequency source and is transferred along a one-dimensional channel from QD1 to QD2. (**d**) The mesoscopic capacitor [20,21,22,23,24,25,26], in which a gate-driven quantum dot emits single electrons through a QPC in a two-dimensional electron gas. This platform will be extensively discussed in this review.

Beyond theoretical interest, this question is important for ongoing experiments with mesoscopic devices aimed towards the full control of single electrons out of equilibrium. Figure 1 reports some of these experiments [8,9,10,11,12,13,14,15,16,17,18,19], in addition to the mesoscopic capacitor [20,21,22,23,24,25,26], which will be extensively discussed in this review. These experiments and significant others [27,28,29,30,31,32,33] have a common working principle: A fast [34] time-dependent voltage drive V(t), applied either on metallic or gating contacts, triggers emission of well defined electronic excitations. Remarkably, these experiments achieved to generate, manipulate, and detect single electrons on top of a complex many-body state such as the Fermi sea. A comprehensive review of these experiments can be found in Ref. [35].

In this context, interactions are usually considered detrimental, as they are responsible for inelastic effects leading to diffusion and dephasing [36]. Interaction screening or, alternatively, the disappearance of such inelastic effects at low driving energies or temperatures [37,38,39,40,41,42] is thus crucial to identify single-electron long-lived excitations (quasi-particles) close to the Fermi surface. The possibility of identifying such excitations, even in the presence of strong Coulomb interactions, is the core of the Fermi liquid theory of electron gases in solids [43,44], usually identified with the $\propto T^{2}$ suppression of resistivities in bulk metals. It is the validity of this theory for conventional metals that actually underpins the success of Landauer–Büttiker elastic scattering theory [45,46,47] to describe coherent transport in mesoscopic devices.

The aim of this review is to show how a similar approach can also be devised to describe transport in mesoscopic conductors involving the interaction of artificial quantum impurities. In these systems, electron-electron interactions are only significant in the confined and local quantum dot regions, and not in the leads for instance, therefore we use the terminology of a Local Fermi Liquid theory (LFL) in contrast to the conventional Fermi liquid approach for bulk interactions. Originally, the first LFL approach [48] was introduced to derive the low energy thermodynamic and transport properties of Kondo local scatterers in materials doped with magnetic impurities [49]. In this review, we will show how LFLs provide the unifying framework to describe both elastic scattering and strong correlation phenomena in the out-of-equilibrium dynamics of mesoscopic devices. This approach makes also clear how inelastic effects, induced by Coulomb interactions, become visible and unavoidable as soon as such systems are strongly driven out of equilibrium. We will discuss how extensions of LFLs and related approaches describe such regimes as well.

**Figure 2:**
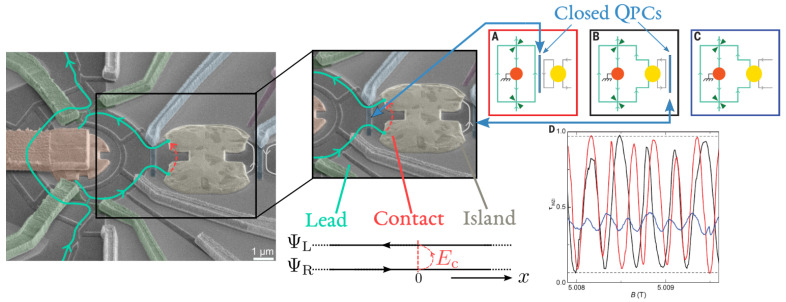
Left—Mach–Zehnder interferometer with a floating metallic island (colored in yellow) [50]. The green lines denote chiral quantum Hall edge states, which can enter the floating island passing through a gate-tunable QPC (in blue). An additional QPC separates the floating island from an additional reservoir on its right. Center—The floating island is described by two infinite counter-propagating edges, exchanging electrons coherently thanks to the charging energy $E_{c}$ of the island (red arrow). Right—Mach–Zehnder visibility of the device as a function of magnetic field *B*. Oscillation of this quantity as function of *B* signals quantum coherent interference between two paths encircling an Aharonov–Bohm flux. In the situation sketched in box A, the first QPC is closed and the interferometer is disconnected from the island and visibility oscillations are observed, as expected (red line). Remarkably, the oscillations persist (black line) in the situation sketched in box B, where the leftmost QPC is open and one edge channel enters the floating island. The visibility oscillations are only suppressed in the situation sketched in box C, where the rightmost QPC is also open and the island is connected to a further reservoir (blue line).

As a paradigmatic example, we will focus on recent experiments showing the electron transfer with Coulomb interactions [50], (see Figure 2), and, in more detail, on the mesoscopic capacitor [20,21,22,23,24,25,26], (see Figure 6). The mesoscopic capacitor does not support the DC transport, and it makes possible the direct investigation and control of the coherent dynamics of charge carriers. The LFL description of such devices entails the seminal results relying on self-consistent elastic scattering approaches by Büttiker and collaborators [51,52,53,54,55,56], but it also allows one to describe effects induced by strong Coulomb correlations, which remain nevertheless elastic and coherent. The intuition provided by the LFL approach is a powerful lens through which it is possible to explore various out-of-equilibrium phenomena, which are coherent in nature but are governed by Coulomb interactions. As an example, we will show how a bold treatment of Coulomb interaction unveils originally overlooked strong dynamical effects, triggered by interactions, in past experimental measurements showing fractionalization effects in out-of-equilibrium charge emission from a driven mesoscopic capacitor [25].

This review is structured as follows. In Section 2, we give a simple example showing how Coulomb interactions trigger phase-coherent electron state transfer in experiments as those reported in Ref. [50], Figure 2. Section 3 discusses how the effective LFL approach [57,58,59,60,61,62,63,64] provides the unified framework describing such coherent phenomena. In Section 4, we consider the study of the low-energy dynamics of the mesoscopic capacitor, in which the LFL approach has been fruitfully applied [65,66,67,68,69], showing novel quantum coherent effects. Section 5 extends the LFL approach out of equilibrium and describes signatures of interactions in measurements of strongly driven mesoscopic capacitors [25].

## 2. Phase-Coherence in Quantum Devices with Local Interactions

To illustrate the restoration of phase coherence at low temperatures in the presence of interactions, we consider two counter-propagating edge states entering a metallic quantum dot, or cavity. Such a system was recently realized as a constitutive element of the Mach–Zender interferometer of Ref. [50], reported in Figure 2. In that experiment, the observation of fully preserved Mach–Zehnder oscillations, in a system in which a quantum Hall edge state penetrates a metallic floating island demonstrates an interaction-induced, restored phase coherence [70,71].

The dominant electron-electron interactions in the cavity have the form of a charging energy [1,2,3]

(1) $$H_{c}=E_{c}{\left[N-N_{g}\left(t\right)\right]}^{2}\;,$$

in which *N* is the number of electrons in the island, $C_{g}$ is the geometric capacitance, and $N_{g}=C_{g}V_{g}\left(t\right)/e$ is the dimensionless gate voltage, which corresponds to the number of charges that would set in the cavity if *N* was a classical, non-quantized, quantity. We also define the charging energy $E_{c}=e^{2}/2C_{g}$: The energy cost required the addition of one electron in the isolated cavity. For the present discussion, we neglect the time-dependence of the gate-potential $V_{g}$, which will be reintroduced to describe driven settings. In the linear-dispersion approximation, the right/left-moving fermions $\Psi_{R,L}$ in Figure 2, moving with Fermi velocity $v_{F}$, are described by the Hamiltonian:

(2) $$H_{\mathrm{kin}}=v_{F}\hbar \sum_{\alpha =R/L}\int_{-\infty }^{\infty }dx\;\Psi_{\alpha }^{†}\left(x\right)\left(-i\alpha \partial_{x}\right)\Psi_{\alpha }\left(x\right)\;,$$

with the sign α=+/− multiplying the $\partial_{x}$ operator for right- and left-movers respectively. The floating island occupies the semi-infinite one-dimensional space located at x>0 with the corresponding charge $N=\sum_{\alpha }\int_{0}^{\infty }dx\Psi_{\alpha }^{†}\left(x\right)\Psi_{\alpha }\left(x\right)$. It is important to stress that the model (1)–(2) is general and effective in describing different quantum dot devices. It was originally suggested by Matveev to describe quantum dots connected to leads through a single conduction channel [72] and it equally describes the mesoscopic capacitor, see Section 4 and Section 5.

The model (1)–(2) characterizes an open-dot limit in the sense that it does not contain an explicit backscattering term coupling the *L* and *R* channels. It can be solved exactly by relying on the bosonization formalism [73,74,75,76], which, in this specific case, maps interacting fermions onto non-interacting bosons [72,77,78]. Using this mapping, one can show that the charging energy $E_{c}$ perfectly converts, far from the contact, right-movers into left-movers. This fact is made apparent by the “reflection” Green function $G_{\mathrm{LR}}$ [77]:

(3) $$\begin{array}{c} G_{\mathrm{LR}}=\langle T_{\tau }\Psi_{L}^{†}\left(x,\tau \right)\Psi_{R}\left(x^{\prime },0\right)\rangle ≃e^{-i2\pi N_{g}}\frac{T/2v_{F}}{\sin\left[\frac{\pi T}{\hbar }\left(\tau +i\left(x+x^{\prime }\right)-i\frac{\pi \hbar }{E_{c}e^{C}}\right)\right]}\;, \end{array}$$

which we consider at finite temperature *T*. In Equation (3), $T_{\tau }$ is the usual time-ordering operator defined as $T_{\tau }A\left(\tau \right)B\left(\tau^{\prime }\right)=\theta \left(\tau -\tau^{\prime }\right)A\left(\tau \right)B\left(\tau^{\prime }\right)\pm \theta \left(\tau^{\prime }-\tau \right)B\left(\tau^{\prime }\right)A\left(\tau \right)$, in which the sign +/− is chosen depending on the bosonic/fermionic statistics of the operators *A* and *B* [79] and θ(τ) is the Heaviside step function. As first noted by Aleiner and Glazman [77], the form of $G_{\mathrm{LR}}$ at large (imaginary) time τ corresponds to the elastic reflection of the electrons incident on the dot, with a well-defined scattering phase $\pi N_{g}$. The correlation function (3) would be identical if the interacting dot was replaced with a non-interacting wire of length $v_{F}\pi \hbar /E_{c}\gamma$ (with lnγ=C≃0.5772 being Euler’s constant), imprinting a phase $\pi N_{g}$ when electrons are back-reflected at the end of the wire [80].

The physical picture behind Equation (3) is that an electron entering and thereby charging the island violates energy conservation at low temperature and must escape on a time scale $\hbar /E_{c}$ fixed by the uncertainty principle. The release of this incoming electron can happen either elastically, in which case the electron keeps its energy, or inelastically via the excitation of electron-hole pairs. As we discuss in Section 3.1, inelastic processes are suppressed by the phase space factor ${\left(\varepsilon /E_{c}\right)}^{2}$, ε being the energy of the incoming electron, and they die out at low energy or large distance (time), reestablishing purely elastic scattering despite a nominally strong interaction.

**Figure 3:**
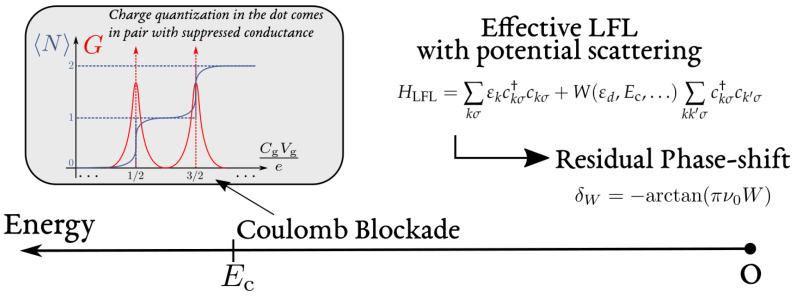
Coulomb blockade and emergent LFL behavior. When the typical energy of the system (temperature, bias-voltage, …) is smaller than the charging energy $E_{c}$, charge quantization $Q=e\langle N\rangle$ in the dot suppresses the conductance *G* of the system. Degeneracy between different charge occupations lead to conductance peaks, which become larger the stronger the tunnel exchange of electrons with the leads. Conductance peaks and charge quantization disappear in the open-dot limit. For any tunneling strength, the dot behaves as an elastic scatterer described by the LFL theory (8), with potential scattering of strength *W*, inducing a phase-shift $\delta_{W}$ on lead electrons set by the dot occupation 〈N〉.

Equation (3) is thus a remarkable example of how interactions trigger coherent effects in mesoscopic devices. It has been derived here for an open dot, a specific limit in which the charge quantization of the island is fully suppressed. However, the restoration of phase coherence at low energy is more general and applies for an arbitrary lead-island transmission, in particular in the tunneling limit where the charge states of the quantum dot are well quantized [1,2,3]. This quantization is known to induce Coulomb blockade in the conductance of the device, see Figure 3. Nevertheless, a Coulomb blockaded dot acts at low energy as an elastic scatterer imprinting a phase δ [81,82] related to its average occupation $\langle N\rangle$ via the Friedel sum rule, see Section 3.2. For weak transmissions, $\langle N\rangle$ strongly deviates from the classical value $N_{g}$. These features constitute the main characteristics of the local Fermi liquid picture detailed in the forthcoming sections.

## 3. What Are Local Fermi Liquids and Why Are They Important to Understand Quantum-Dot Devices?

In this Section, we introduce the local Fermi liquid theory and discuss its application to quantum transport devices. The general system considered in this paper is a central interacting region, such as a quantum dot, connected to leads described as non-interacting electronic reservoirs. The Hamiltonian takes the general form:

(4) $$H=H_{\mathrm{res}}+H_{\mathrm{res}-\mathrm{dot}}+H_{\mathrm{dot}}+H_{c}\;.$$

The first term describes the lead reservoir, which could be either a normal metal [14], a chiral edge state in the quantum Hall regime [22,29], or a superconductor [18]. In the case of a normal metal, it is given by:

(5) $$H_{\mathrm{res}}=\sum_{k}\varepsilon_{k}c_{k}^{†}c_{k}\;,$$

in which $c_{k}$ annihilates a fermion in the eigenstate state *k* of energy $\varepsilon_{k}$ in the reservoir. For instance, in Figure 2, the reservoir modes correspond to the x<0 components of the operators $\Psi_{R/L}$. The field $\Psi_{\mathrm{res}}\left(x\right)=\theta \left(-x\right)\Psi_{R}\left(x\right)+\theta \left(x\right)\Psi_{L}\left(-x\right)$, with θ(x) the Heaviside step function, unfolds the chiral field onto the interval x∈[−∞,∞] and its Fourier transform $c_{k}=\int_{-\infty }^{\infty }dxe^{-ikx}\Psi_{\mathrm{res}}\left(x\right)$ recovers Equation (5) from Equation (2), with $\varepsilon_{k}=\hbar v_{F}k$.

The single particle physics of the quantum dot is described instead by:

(6) $$H_{\mathrm{dot}}=\sum_{l}\left(\varepsilon_{d}+\varepsilon_{l}\right)n_{l}$$

in which $n_{l}=d_{l}^{†}d_{l}$ counts the occupation of the orbital level *l* and $d_{l}$ annihilates fermions in that state. The spectrum can be either discrete for a finite size quantum dot or dense for a metallic dot as in the case of Figure 2. We also introduced the orbital energy $\varepsilon_{d}$ as a reference. $H_{\mathrm{res}-\mathrm{dot}}$ describes the exchange of electrons between dot and reservoir. It has generally the form of a tunneling Hamiltonian:

(7) $$H_{\mathrm{res}-\mathrm{dot}}=t\sum_{k,l}\left[c_{k}^{†}d_{l}+d_{l}^{†}c_{k}\right]\;,$$

in which we neglect, for simplicity, any *k* dependence of the tunneling amplitude *t*. The charging energy $H_{c}$ is given in Equation (1) with the dot occupation operator $N=\sum_{l}n_{l}$.

**Figure 4:**
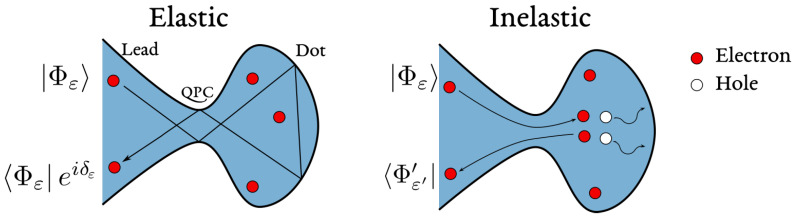
Difference between elastic (**left**) and inelastic (**right**) events for electrons scattering on a quantum dot. In the elastic case, electrons do not change energy ε. The wave function is preserved and the only residual effect of scattering is a phase-shift $\delta_{\varepsilon }$. In the inelastic case, many-body interactions trigger the creation of particle-hole pairs. Outgoing electrons are then emitted in a state $|\Phi_{\varepsilon^{\prime }}^{\prime }\rangle$ of energy $\varepsilon^{\prime }$ different from the initial ε and phase coherence is gradually lost.

Without any approximation, deriving the out-of-equilibrium dynamics of interacting models such as Equation (4) is a formidable task. The presence of local interactions leads to inelastic scattering events, creating particle-hole pairs when electron scatter on the dot (see Figure 4). From a technical point of view, such processes are difficult to handle and, even if these difficulties are overcome, one has to identify the dominant physical mechanisms governing the charge dynamics. In our discussion, interactions are usually controlled by the charging energy $E_{c}$, which cannot be treated perturbatively in Coulomb blockade regimes. The possibility to rely on Wick’s theorem [83], when performing perturbative calculations in the exchange term $H_{\mathrm{res}-\mathrm{dot}}$, is also denied. Thus, one has to look for a more efficient theoretical approach.

### 3.1. The Local Fermi Liquid

The local Fermi liquid approach is justified by the physical picture already presented in Section 2, namely that an incoming reservoir electron with an energy much smaller than the charging energy of the quantum dot is effectively scattered in a purely elastic way [77]. At temperatures well below the charging energy $E_{c}$, energy conservation prevents any permanent change in the charge of the quantum dot and each electron entering the dot must be compensated by an electron leaving it within the (short) time $\hbar /E_{c}$ fixed by the uncertainty principle. The electron escape can occur via elastic or inelastic processes, sketched in Figure 4, depending on whether the electron energy is preserved or not. Inelastic processes cause decoherence and call for a many-body approach to be properly evaluated.

At low energy ε of the incoming electron, the inelastic processes are typically suppressed by the ratio ${\left(\varepsilon /E_{\mathrm{FL}}\right)}^{2}$ [43,44]. $E_{\mathrm{FL}}$ is a Fermi liquid energy scale, typically of the order of the charging energy $E_{c}$. Nevertheless, in the presence of spin-fluctuations, the emergence of strong Kondo correlations, to be discussed in Section 3.3.2, can sensibly reduce $E_{\mathrm{FL}}$ down to the Kondo energy scale $T_{K}$, (see Equation (26)). Therefore, in the ε→0 limit, inelastic processes are ignored and the scattering is purely elastic. It is described within a single-particle formalism where the scattering by the quantum dot imprints a phase shift $\delta_{W}$ to the outgoing electronic wave functions. This phase shift alone incorporates all interaction and correlation effects.

The simplest model entailing these features is a free Fermi gas in which a delta barrier located at x=0 (the entrance of the dot) scatters elastically quasi-particles. In the language of second quantization, the delta barrier is described by the electron operator $\Psi_{\mathrm{res}}^{†}\left(x=0\right)\Psi_{\mathrm{res}}\left(x=0\right)$, and its strength *W* has to depend on the parameters of the parent model (such as the orbital energy $\varepsilon_{d}$, the charging energy $E_{c}$, etc.). Switching to momentum space, such model is a free Fermi gas with a potential scattering term:

(8) $$H_{\mathrm{LFL}}=\sum_{k\sigma }\varepsilon_{k}c_{k\sigma }^{†}c_{k\sigma }+W\left(\varepsilon_{d},E_{c},\ldots \right)\sum_{kk^{\prime }\sigma }c_{k\sigma }^{†}c_{k^{\prime }\sigma }+O{\left(\frac{\varepsilon }{E_{\mathrm{FL}}}\right)}^{2}\;$$

where the scattering potential leads, as shown in Appendix A, to the quasi-particle phase shift:

(9) $$\delta_{W}=-\arctan\left(\pi \nu_{0}W\right)\;,$$

in which $\nu_{0}$ is the density of states of the lead electrons at the Fermi energy, see also Equation (A21) in Appendix A for its rigorous definition. In this *Local Fermi Liquid* Hamiltonian, σ labels either a spin polarization or a channel. The number of channels in the lead can be controlled by the opening of a quantum point contact [84]. The potential strength *W* can be cumbersome to compute, but it is nevertheless related to the occupancy of the quantum dot via the Friedel sum rule, as explained in Section 3.2.

The simplicity of the local Fermi liquid Hamiltonian (8) makes it powerful to evaluate low energy properties. Being non-interacting, it also includes the restoration of phase coherence in the scattering of electrons seen in Section 2. An important assumption that we made is that the system exhibits a Fermi liquid ground state, or Fermi liquid fixed point in the language of the renormalization group. Non-Fermi liquid fixed points exist and cannot be described by such Hamiltonian [85], but they are generally fine-tuned and unstable with respect to perturbations. Furthermore, Equation (8) is not applicable to genuine out-of-equilibrium regimes, when the perturbations are too strong or vary too fast with respect to the Fermi liquid energy scale $E_{\mathrm{FL}}$ (typically of the order of the charging energy $E_{c}$).

### 3.2. The Role of the Friedel Sum Rule in Local Fermi Liquid Theories

In the process of electron backscattering by the quantum dot, or by the interacting central region, the phase shift relates the incoming and outgoing electronic wavefunctions $\Psi_{L}\left(0^{-}\right)=e^{2i\delta_{\varepsilon }}\Psi_{R}\left(0^{-}\right)$, see Appendix A for an explicit illustration on the resonant level non-interacting model. The Friedel sum rule [86] establishes the relation between the average charge occupation of the dot $\langle N\rangle$ and this phase-shift δ. Its form in the case of *M* conducting channels reads:

(10) $$\langle N\rangle =\frac{1}{\pi }\sum_{\sigma =1}^{M}\delta_{\sigma }\;.$$

The Friedel sum rule has been proven rigorously for interacting models [87,88]. It is valid as long the ground state has a Fermi liquid character. Physically, it can be understood in the following way: The derivative of the phase shift $\delta_{\varepsilon }$ with respect to energy defines (up to *ℏ*) the Wigner-Smith scattering time [89], see Equation (A38), that is the time delay experienced by a scattered electron. In the presence of a continuous flow of electrons, a time delay implies that some fraction of the electronic charge has been (pumped) deposited in the quantum dot [51,90]. Therefore the phase shift amounts to a left-over charge and it does not matter that electrons are interacting on the quantum dot as long as they are not in the leads, which is the essence of the local Fermi liquid approach.

The Friedel sum rule (10) combined with Equation (9) relates the dot occupancy to the potential scattering strength. For the single-channel case (M=1), one finds:

(11) $$\langle N\rangle =-\frac{1}{\pi }\arctan\left(\pi \nu_{0}W\right)\;.$$

This is an important result because the dot occupation $\langle N\rangle$ is a thermodynamic quantity, which can be also accessed in interacting models, allowing us to address quantitatively the close-to-equilibrium dynamics of driven settings, as we will discuss in Section 4.5.

We emphasize that the local Fermi liquid approach of Equation (8) can be extended to perturbatively include inelastic scattering and higher-order energy corrections and relate these terms to thermodynamic observables. This program has been realized in detail for the Anderson and Kondo models [57,58,59,60,61,62,63,64,91].

### 3.3. Derivation of the LFL Theory in the Coulomb Blockade and Anderson Model

We show now how the effective theory (8) can be explicitly derived from realistic models describing Coulomb blockaded quantum dot devices [66]. We focus on the Coulomb Blockade Model (CBM) [2,3]:

(12) $$H_{\mathrm{CBM}}=\sum_{k}\varepsilon_{k}c_{k}^{†}c_{k}+t\sum_{k,l}\left[c_{k}^{†}d_{l}+d_{l}^{†}c_{k}\right]+\sum_{l}\left(\varepsilon_{d}+\varepsilon_{l}\right)d_{l}^{†}d_{l}+E_{c}{\left(N-N_{g}\right)}^{2}\;$$

and the Anderson Impurity Model (AIM), which, in its standard form, reads [92,93]:

(13) $$H_{\mathrm{AIM}}=\sum_{k,\sigma }\varepsilon_{k,\sigma }c_{k,\sigma }^{†}c_{k,\sigma }+t\sum_{k,\sigma }\left[c_{k,\sigma }^{†}d_{\sigma }+d_{\sigma }^{†}c_{k,\sigma }\right]+\varepsilon_{d}\sum_{\sigma }d_{\sigma }^{†}d_{\sigma }+Un_{↑}n_{↓}\;.$$

Adding $-eV_{g}N+E_{c}N_{g}^{2}$ to the AIM and for $U=E_{c}$, the charging energy (1) becomes apparent in Equation (13), as in Equations (4)–(12). The CBM coincides with the Hamiltonian (4) and describes the mesoscopic capacitor in the Quantum Hall regime: A reservoir of spinless fermions $c_{k}$ of momentum *k* is tunnel coupled to an island with discrete spectrum $\varepsilon_{l}$. The AIM includes the spin degree of freedom and considers a single interacting level in the quantum dot. This model encompasses Kondo correlated regimes [49,94] and describes well the experiments [95,96].

To derive the LFL Hamiltonian (8), we rely on the Schrieffer–Wolff (SW) transformation [44,97], first devised to map the AIM [94] onto the Coqblin–Schrieffer model [98], and that we extend here to the CBM. Far from the charge degeneracy points, in the $t\ll E_{c}$ limit, the ground-state charge configuration $n=\langle N\rangle$ is fixed by the gate potential $V_{g}$ and fluctuations to n±1 require energies of order $E_{c}$. For temperatures much lower than $E_{c}$, the charge degree of freedom of the quantum dot is frozen, acting but virtually on the low energy behavior of the system. The SW transformation is a controlled procedure to diagonalize perturbatively in *t* the Hamiltonian. The Hamiltonian is separated in two parts $H=H_{0}+H_{\mathrm{res}-\mathrm{dot}}$, in which $H_{0}$ is diagonal in the charge sectors labeled by the eigenvalues *n* of the dot occupation *N*, which are mixed by the tunneling Hamiltonian $H_{\mathrm{res}-\mathrm{dot}}$, involving the tunneling amplitude *t*. The perturbative diagonalization consists of finding the Hermitian operator *S* (of order *t*) generating the unitary $U=e^{iS}$ rotating the Hamiltonian in the diagonal form $H^{\prime }=U^{†}HU$. To leading order in *S* we find:

(14) $$H^{\prime }=H_{0}+H_{\mathrm{res}-\mathrm{dot}}+i\left[S,H_{0}\right]+O\left(t^{2}\right).$$

This Hamiltonian is block diagonal if the condition:

(15) $$iH_{\mathrm{res}-\mathrm{dot}}=\left[S,H_{0}\right]$$

is fulfilled and Equation (14) becomes:

(16) $$H^{\prime }=H_{0}+\frac{i}{2}\left[S,H_{\mathrm{res}-\mathrm{dot}}\right],$$

which is then projected on separated charge sectors.

#### 3.3.1. Coulomb Blockade Model

To derive the effective low-energy form of the CBM model, it is useful, following Grabert [99,100], to decouple the charge occupancy of the dot from the fermionic degree of freedom of the electrons. This is achieved by adding the operator $\hat{n}=\sum_{n}\left|n\rangle \langle n\right|$, measuring to the dot occupation number. The fermionic operators $d_{l}$ in Equation (12) are replaced by new operators describing a non-interacting electron gas in the dot. The Hamiltonian (12) acquires then the form:

(17) $$H_{\mathrm{CBM}}=\sum_{k}\varepsilon_{k}c_{k}^{†}c_{k}+t\sum_{n,k,l}\left[d_{l}^{†}c_{k}|n+1\rangle \langle n|+h.c.\right]+\sum_{l}\varepsilon_{l}d_{l}^{†}d_{l}+\varepsilon_{d}\hat{n}+E_{c}{\left(\hat{n}-N_{g}\right)}^{2}\;.$$

The operator $S=s+s^{†}$ fulfilling the condition (15) reads:

(18) $$\begin{array}{cccc} s= & it\sum_{k,l,n}s_{kln}c_{k}^{†}d_{l}|n-1\rangle \langle n|, & s_{kln}= & \frac{1}{\varepsilon_{l}-\varepsilon_{k}+E_{c}\left(2n-1\right)+\varepsilon_{d}}. \end{array}$$

This operator, when inserted into Equation (16), also generates higher order couplings between sectors of charge *n* and n±2, which we neglect in the present discussion. The Hamiltonian becomes then block diagonal in the sectors given by different values of *n*. For $\left(N_{g}-\varepsilon_{d}C_{g}/e\right)\in \left[-1/2,1/2\right]$, the lowest energy sector corresponds to n=0 and the effective Hamiltonian reads $H_{\mathrm{CBM}}^{\prime }=H_{0}+H_{B}$, with:

(19) $$H_{B}=\frac{t^{2}}{2}\sum_{kk^{\prime }ll^{\prime }}\left(s_{kl0}d_{l^{\prime }}^{†}c_{k^{\prime }}c_{k}^{†}d_{l}-s_{kl1}c_{k}^{†}d_{l}d_{l^{\prime }}^{†}c_{k^{\prime }}+h.c.\right).$$

This interaction can be simplified by a mean-field treatment:

(20) $$d_{l}^{†}c_{k}c_{k^{\prime }}^{†}d_{l^{\prime }}=\langle d_{l}^{†}d_{l^{\prime }}\rangle c_{k}c_{k^{\prime }}^{†}+\langle c_{k}c_{k^{\prime }}^{†}\rangle d_{l}^{†}d_{l^{\prime }}=\delta_{ll^{\prime }}\theta \left(-\varepsilon_{l}\right)c_{k}c_{k^{\prime }}^{†}+\delta_{kk^{\prime }}\theta \left(\varepsilon_{k}\right)d_{l}^{†}d_{l^{\prime }}\;,$$

allowing to carry out part of the sums in Equation (19). Notice that the orbital energy $\varepsilon_{d}$ does not appear in Equation (20) as it is now only associated to the charge degree of freedom *n*, while the Fermi gases corresponding to $c_{k}$ and $d_{l}$ have the same Fermi energy $E_{F}=0$. One thus finds the effective low energy model, which, to leading order, reads [66]:

(21) $$H_{\mathrm{CBM}}^{\prime }=H_{0}+\frac{g}{\nu_{0}}\ln\left(\frac{E_{c}-\varepsilon_{d}}{E_{c}+\varepsilon_{d}}\right)\left[\sum_{ll^{\prime }}d_{l}^{†}d_{l^{\prime }}-\sum_{kk^{\prime }}c_{k}^{†}c_{k^{\prime }}\right]\;$$

in which we have introduced the dimensionless conductance $g={\left(\nu_{0}t\right)}^{2}$, corresponding to the conductance of the Quantum Point Contact (QPC) connecting dot and lead in units of $e^{2}/h$. This Hamiltonian describes two decoupled Fermi gases, but affected by potential scattering with opposite amplitudes. Equation (21) coincides with the LFL Hamiltonian (8) for the lead electrons. The phase-shift $\delta_{W}$ (9) allows for the calculation of the charge occupation of the dot to leading order by applying the Friedel sum rule (10):

(22) $$\langle N\rangle =\frac{\delta_{W}}{\pi }=g\;\ln\left(\frac{E_{c}-\varepsilon_{d}}{E_{c}+\varepsilon_{d}}\right).$$

This result reproduces the direct calculation of the dot occupation [99,100,101], showing the validity of the LFL model (8), with Friedel sum rule for the CBM. The extension to *M* channels is obtained by replacing $g\to M{\left(\nu_{0}t\right)}^{2}$ in Equation (22). The extended proof to next-to-leading order in *g* is given in Ref. [66].

#### 3.3.2. Anderson Impurity Model

**Figure 5:**
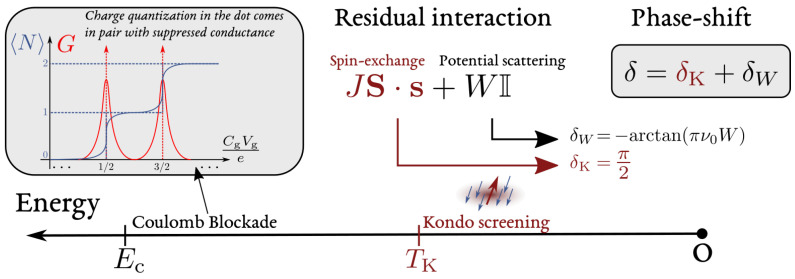
Modification of the physical scenario of Figure 3 in the presence of spin-exchange interactions between the dot and lead electrons in the Anderson Impurity Model (AIM). Spin-exchange interactions trigger the formation of the Kondo singlet below the Kondo temperature $T_{K}$, which is responsible for an additional elastic $\delta_{K}=\pi /2$ phase-shift of lead electrons in the effective Local Fermi Liquid (LFL) theory.

Considering the internal spin degree of freedom in the AIM (13), it does not fundamentally affect the effective LFL behavior. Nevertheless, in the case where a single electron is trapped in the quantum dot ($-U\ll \varepsilon_{d}\ll 0$), the derivation of Equation (8) is more involved, and sketched in Figure 5. The SW transformation maps the AIM onto a Kondo Hamiltonian including a potential scattering term [94]:

(23) $$H_{\mathrm{AM}}^{\prime }=H_{0}+JS\cdot s+W\sum_{kk^{\prime }\sigma }c_{k\sigma }^{†}c_{k^{\prime }\sigma }\;.$$

The spin of the electron in the quantum dot S is coupled anti-ferromagnetically to the local spin of the lead electrons $s=\sum_{kk^{\prime }\tau \tau^{\prime }}c_{k\tau }^{†}\frac{\sigma_{\tau \tau^{\prime }}}{2}c_{k^{\prime }\tau^{\prime }}$, with $\sigma_{\tau \tau^{\prime }}$ the vector composed of the Pauli matrices, and

(24) $$\begin{array}{cccc} J & =\frac{2\Gamma }{\pi \nu_{0}}\left(\frac{1}{\varepsilon_{d}+U}-\frac{1}{\varepsilon_{d}}\right)\;, & W & =-\frac{\Gamma }{2\pi \nu_{0}}\left(\frac{1}{\varepsilon_{d}+U}+\frac{1}{\varepsilon_{d}}\right)\;, \end{array}$$

in which we introduced the hybridization energy $\Gamma =\pi \nu_{0}t^{2}$, corresponding to the width acquired by the orbital level when coupled to the lead and which depends on the density of states of the lead electrons at the Fermi energy $\nu_{0}$, see also Equation (A21) in Appendix A for its rigorous definition. Neglecting for the moment the Kondo anti-ferromagnetic coupling controlled by *J*, the LFL Hamiltonian (8) is directly recovered. Nevertheless, the potential scattering term is absent (W=0) at the particle-hole symmetric point $\varepsilon_{d}=-U/2$. At this point, the charge on the dot is fixed to one by symmetry, and the absence of potential scattering allows to derive various rigorous results, for instance concerning the ground state properties relying on Bethe ansatz [102,103]. It is a well-established fact that the system described by Equation (23) behaves as a LFL at low energies [48,104,105] and that the Friedel sum rule applies [87]. As a consequence, the Kondo coupling is responsible for the phase-shift of the low energy quasi-particles. Particle-hole symmetry, spin degeneracy, and Friedel sum rule fix the Kondo phase-shift to $\delta_{K}=\pi /2$. The Friedel sum rule states that:

(25) $$\langle N\rangle =2\frac{\delta_{K}}{\pi }\;,$$

$\langle N\rangle =1$ because of particle-hole symmetry and the factor 2 signals spin degeneracy, fixing $\delta_{K}=\pi /2$. The detailed description of the Kondo effect is far beyond the scope of this review and we direct the interested reader to Ref. [49] for a comprehensive review and to Refs. [106,107,108,109,110,111] for the description of the low energy fixed point relying on boundary conformal field theory. For the scopes of this review it is enough to mention that below the Kondo temperature: [102,103,112,113]

(26) $$T_{K}=\frac{e^{\frac{1}{4}}\gamma }{2\pi }\sqrt{\frac{2U\Gamma }{\pi }}e^{\frac{\pi \epsilon_{d}\left(\epsilon_{d}+U\right)}{2U\Gamma }}\;,$$

the spin-exchange coupling *J* in Equation (23) flows to infinity in the renormalization group sense. The relevance of this interaction brings the itinerant electrons to screen the local spin-degree of freedom of the quantum dot and phase-shifts the resulting quasi-particles by $\delta_{K}$, see Figure 5. The phase-shift π/2 acquires thus a simple interpretation in one dimension [44,106]: Writing $\Psi_{R}=e^{2i\delta }\Psi_{L}$ for a given spin channel at the impurity site, $\delta =\delta_{K}=\pi /2$ leads to $\Psi_{R}+\Psi_{L}=0$. The fact that the wave-function is zero at the impurity site, corresponds to the situation in which an electron screens the impurity spin, leading to Pauli blockade (due to Pauli principle), thus preventing other electrons with the same spin to access the impurity site. This dynamical screening of the impurity spin forms the so called “Kondo cloud” [114,115,116,117], see Figure 5. It is responsible for increasing the local density of states and leads to the Abrikosov–Suhl resonance [118], which causes the increase, below $T_{K}$, of the dot conductance in Coulomb blockaded regimes [93,119,120]. Remarkably, the Kondo phase-shift $\delta_{K}=\pi /2$ and the Kondo screening cloud have been also directly observed in two recent distinct experiments [121,122]. In Section 4.7, we illustrate how such phenomena also affect the dynamical properties of the mesoscopic capacitor in a non-trivial way.

It remains to establish the combined role of spin-exchange and potential scattering on the low-energy quasi-particles. Remarkably, the phase-shift $\delta_{W}$, caused by potential scattering, is additive to $\delta_{K}$ [123,124,125]:

(27) $$\delta =\delta_{K}+\delta_{W},$$

and can thus be calculated independently. The validity of the above expression is demonstrated by comparison with the exact Bethe ansatz solution of the AIM [102,103]. Inserting the expression (9) for the phase-shift caused by the potential scattering in the Friedel sum rule one finds:

(28) $$\langle N\rangle =\frac{2}{\pi }\left[\delta_{K}-\arctan\left(\pi \nu_{0}W\right)\right]=1+\frac{\Gamma }{\pi }\left(\frac{1}{\varepsilon_{d}+U}+\frac{1}{\varepsilon_{d}}\right)\;.$$

This expression is consistent with the condition $\langle N\rangle =1$, imposed by particle-hole symmetry, but also with the static charge susceptibility $\chi_{c}$, which was derived with the Bethe ansatz [126]:

(29) $$\chi_{c}={\left.-\frac{\partial \langle N\rangle }{\partial \varepsilon_{d}}\right|}_{\varepsilon_{d}=-\frac{U}{2}}=\frac{8\Gamma }{\pi U^{2}}\left(1+\frac{12\Gamma }{\pi U}\;+\ldots \right).$$

The extension of this proof to next-to-leading order in *t*, is given in Refs. [66,68] and it shows how LFL approaches are effective in providing analytic predictions out of particle-hole symmetry as well, extending Bethe ansatz results.

This discussion concludes our demonstration of the persistence of elastic and coherent effects triggered by interactions at equilibrium. Local Fermi liquids provide a general framework to describe interacting and non-interacting systems at low energy, within an effective elastic scattering theory. Nevertheless, it is important to stress that LFL theories can fail in specific cases, such as overscreened Kondo impurities [127,128], and that their validity is limited to close-to-equilibrium/low-energy limits. It is thus expected that interactions become crucial as soon as such systems are driven out of equilibrium. We will illustrate now how the LFL theory allows to describe exotic, but still coherent in nature, dynamical effects in a paradigmatic setup such as the mesoscopic capacitor.

## 4. The Mesoscopic Capacitor

**Figure 6:**
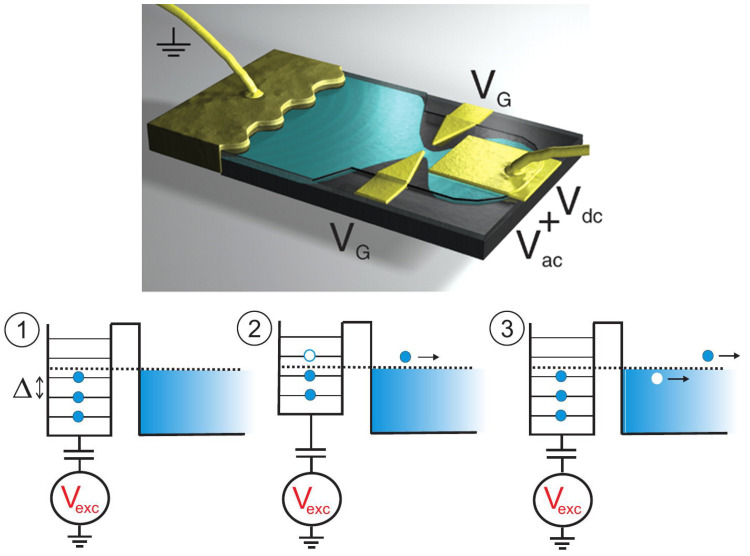
Top—First realization of the mesoscopic capacitor [20]: A two-dimensional electron gas (2DEG) in the Quantum Hall regime is coupled to a quantum cavity via a gate-controlled QPC. Bottom—Working principle of a single electron emission [22,23,24,25,26]. A gate potential moves the quantized levels of the cavity above and below the Fermi surface of the coupled reservoir. Electron/hole emission in steps 2 and 3 follows from moving occupied/empty orbitals above/below the Fermi surface.

The mesoscopic capacitor in Figure 6 plays a central role in the quest to achieve full control of scalable coherent quantum systems [4,129,130]. A mesoscopic capacitor is an electron cavity coupled to a lead via a QPC and capacitively coupled to a metallic gate [51,52,53]. The interest in this device stems from the absence of DC transport, making possible the investigation and control of the coherent dynamics of single electrons. The first experimental realization of this system was a two-dimensional cavity in the quantum Hall regime [20,21], exchanging electrons with the edge of a bulk two-dimensional electron gas (2DEG). Operated out of equilibrium and in the weak tunneling limit, this system allows the triggered emission of single electrons [22,23,24], and paved the way to the realization of single-electron quantum optics experiments [131,132,133,134], as well as probing electron fractionalization [25,135], accounted by the scattering of charge density waves (plasmons) in the conductor [136,137,138,139,140,141,142,143], and their relaxation [26]. On-demand single-electron sources were also recently realized with real-time switching of tunnel-barriers [27,28,29,30,31,32], electron sound-wave surfing [14,16,144], generation of levitons [8,9,10,11,13], and superconducting turnstiles [18,19]. We direct again the interested reader to Ref. [35] for a comprehensive review of these experiments.

The key question concerning the dynamics of a mesoscopic capacitor is which electronic state, carrying a current I, is emitted from the cavity following a change in the gate voltage $V_{g}$. The linear response is characterized by the admittance A(ω),

(30) $$I\left(\omega \right)=A\left(\omega \right)V_{g}\left(\omega \right)+O\left(V_{g}^{2}\right)\;.$$

**Figure 7:**
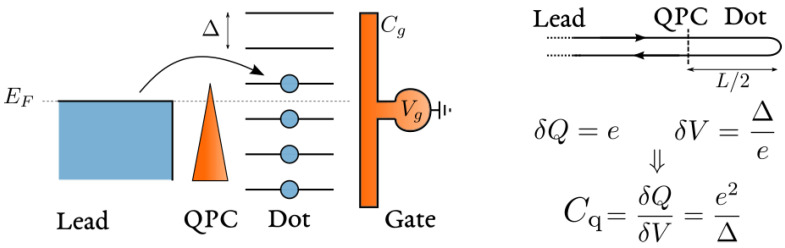
Physical origin of the quantum capacitance $C_{q}$. Pauli exclusion forces electrons entering the dot to pay an energy price equal to the local level spacing Δ, resulting in a capacitance $C_{q}=e^{2}/\Delta$. On the right, a one-dimensional representation of the mesoscopic capacitor, with a dot of size *ℓ*.

In their seminal work, Büttiker and coworkers showed that the low-frequency admittance of a mesoscopic capacitor reproduces the one of a classical *RC* circuit [51,52,53],

(31) $$A\left(\omega \right)=-i\omega C\left(1+i\omega R_{q}C\right)+O\left(\omega^{3}\right)\;,$$

in which both the capacitance *C* and the *charge relaxation resistance* $R_{q}$ probe novel coherent dynamical quantum effects. The capacitance *C* was originally interpreted as an *electro-chemical capacitance* $1/C=1/C_{g}+1/C_{q}$, series of a *geometric* ($C_{g}$) and a *quantum* ($C_{q}$) contribution [21,51,52,53]. The geometric contribution is classical and depends on the shape of the capacitive contact between gate and quantum dot. The quantum contribution is a manifestation of the Pauli exclusion principle and was found proportional to the local density of states in the cavity, see Figure 7. Remarkably, the charge relaxation resistance $R_{q}=h/2e^{2}$ was predicted to be universally equal to half of the resistance quantum in the case of one conducting channel [20], independently of the transparency of the QPC connecting cavity and lead. This result is in striking contrast with the resistance measured in DC experiments and was originally labeled as a *Violation of Kirchhoff’s Laws for a Coherent RC Circuit* [20]. Reference [21] extensively reviews the original theoretical predictions and their experimental confirmation, in a non-interacting and self-consistent setting, which we also review and put in relation with their Hamiltonian formulation in Appendix B.

Below, we discuss how the LFL approach challenges and extends the above studies. In particular:

1. The total capacitance *C* is given by the static charge susceptibility $\chi_{c}=-e^{2}\partial \langle N\rangle /\partial V_{g}$ of the cavity and does not generally correspond to a series of a geometric and quantum contribution, proportional to the density of states in the cavity. For instance, in Kondo regimes, the charge susceptibility of the cavity remains small, because of frozen charge fluctuations, while the density of states increases below the Kondo temperature [49]. This effect was directly probed in a recent experiment with a quantum dot device embedded in circuit-QED architecture [145];
2. A LFL low energy behavior implies universality of the charge relaxation resistance in the single channel case. In particular, the universality of $R_{q}$ stems from a Korringa–Shiba (KS) relation [146]
  (32) Imχc(ω)]ω→0=ωℏπχc2(ω=0),
  in which $\chi_{c}\left(\omega \right)$ is the Fourier transform of the dynamical charge susceptibility (37);
3. The LFL approach shows various non-trivial dissipative effects triggered by strong correlations. In particular, it predicts a mesoscopic crossover between two universal regimes in which $R_{q}=h/2e^{2}\to h/e^{2}$ [65] by increasing the dot size, also at charge degeneracy, in which the CBM maps on the Kondo model [101]. It also predicts giant dissipative regimes, described by giant universal peaks in $R_{q}$, triggered by the destruction of the Kondo singlet by a magnetic field [67,147];
4. In proper out-of-equilibrium regimes, interactions and inelastic effects become unavoidable and circuit analogies, such as Equation (31), do not capture the dynamic behavior of the mesoscopic capacitor [148]. We show here how previously published data [25] also show a previously overlooked signature of non-trivial many-body dynamics induced by interactions.

### 4.1. Hamiltonian Description of the Quantum * RC* Circuit: Differential Capacitance and Korringa–Shiba Relation

Expanding the square in Equation (1) and neglecting constant contributions, $H_{c}$ renormalizes the orbital energy $\varepsilon_{d}$ in Equation (12) and adds a quartic term in the annihilation/creation operators $d_{l}$, namely

(33) $$H_{c}=-eV_{g}\left(t\right)N+E_{c}N^{2}\;.$$

The driving gate voltage $V_{g}$ couples to the charge occupation of the quantum dot $Q=e\langle N\rangle$. In single-electron emitters, one operates on the time dependent voltage drive $V_{g}\left(t\right)$ to bring occupied discrete levels above the Fermi surface and then trigger the emission of charge, see Figure 6. The current of the device is a derivative in time of the charge leaving the quantum dot, the admittance reads then, in the Fourier frequency representation,

(34) $$A\left(\omega \right)=-i\omega \frac{Q(\omega )}{V_{g}\left(\omega \right)}\;.$$

We start by considering small oscillations of amplitude $\varepsilon_{\omega }$ of the gate voltage:

(35) $$V_{g}\left(t\right)=V_{g}+\varepsilon_{\omega }\cos\left(\omega \;t\right)\;.$$

Close to equilibrium, expression (34) is calculated relying on Kubo’s linear response theory [149]

(36) $$A\left(\omega \right)=-i\omega e^{2}\chi_{c}\left(\omega \right)\;,$$

in which $\chi_{c}\left(\omega \right)$ is the Fourier transform of the dynamical charge susceptibility:

(37) $$\chi_{c}\left(t-t^{\prime }\right)=\frac{i}{\hbar }\theta \left(t-t^{\prime }\right){\langle \left[N\left(t\right),N\left(t^{\prime }\right)\right]\rangle }_{0}\;.$$

The notation ${\langle \cdot \rangle }_{0}$ refers to quantum averages performed at equilibrium, i.e., without the driving term $V_{g}\left(t\right)$ in Equation (33). The low frequency expansion of $\chi_{c}\left(\omega \right)$ reads:

(38) A(ω)=−iωe2χc+iImχc(ω)+O(ω2),

where we relied on the fact that the even/odd part of the response function (37) coincide with its real/imaginary part, see Appendix C. We also introduce the static charge susceptibility $\chi_{c}=\chi_{c}\left(\omega =0\right)$. The expansion (38) matches that of a classical RC circuit (31). Identifying term by term, we find the expression of the charge relaxation resistance and, in particular, that the capacitance *C* of the mesoscopic capacitor is actually given by a *differential capacitance* $C_{0}$:

(39) C=C0=e2χc=−e2∂N∂εd=∂Q∂Vg,Rq=1e2χc2Imχc(ω)ωω→0.

The differential capacitance is proportional to the density of states of *charge* excitations on the dot, which, as mentioned above, generally differs from the local density of states in the presence of strong correlations. Equation (39) provides also the general condition for the universal quantization of the charge-relaxation resistance $R_{q}=h/2e^{2}$, namely:

(40) Imχc(ω)ω→0=ℏπωχc2.

Such kind of relation is known as a Korringa–Shiba (KS) relation [146]. The KS relation establishes that the imaginary part of the dynamic charge susceptibility, describing dissipation in the system, is controlled by the static charge fluctuations on the dot, $\chi_{c}$.

Additionally, we mention that the relation (40) also affects the phase-shift of reflected or transmitted light through a mesoscopic system in the Kondo LFL regime [150,151,152]. Such situations have been recently realized with quantum-dot devices embedded in circuit-QED architectures [153,154,155,156,157,158,159], in which the driving input signal can be modeled by an AC potential of the form (35).

### 4.2. The Origin of the Differential Capacitance as a ‘Quantum’ Capacitance as far as Interactions are Neglected

As far as interactions are neglected, the differential capacitance $C_{0}$ is a manifestation of the fermionic statistics of electrons, determined by the Pauli exclusion principle. For this reason it has been originally labeled as a ‘quantum’ capacitance $C_{q}$ [51,52,53]. When an electron is added to the quantum dot, in which energy levels are spaced by Δ, the Pauli exclusion principle does not allow one to fill an occupied energy state, but requires to pay a further energy price Δ, see Figure 7. The capacitance associated to this process is then $C_{q}=\delta Q/\delta V$. For one electron δQ=e and δV=Δ/e. Substituting these two expressions, we recover a uniform quantum capacitance:

(41) $$C_{q}=\frac{e^{2}}{\Delta }\;.$$

This expression establishes that the quantum capacitance is proportional to the density of states in the quantum dot at the Fermi energy $C_{q}=e^{2}N\left(E_{F}\right)$, with $N(E_{F})=1/\Delta$, to be distinguished from $\nu_{0}$, the density of states of the lead electrons.

Additionally, the quantum capacitance is related to the dwell-time spent by electrons in the cavity. The general relation is derived in Appendix B, but it also results from simple estimates. Considering the representation of the mesoscopic capacitor of Figure 7, in the open-dot limit, the time spent by an electron in the cavity coincides with its time of flight $\tau_{f}=\ell /v_{F}$: The ratio between the size of the cavity *ℓ* and its (Fermi) velocity $v_{F}$. The level spacing Δ of an isolated cavity of size *ℓ* is estimated by linearizing the spectrum close to the Fermi level. The distance in momentum between subsequent levels is h/ℓ, corresponding to $\Delta =hv_{F}/\ell =h/\tau_{f}$. Substituting in Equation (41) leads to an equivalent expression for the quantum capacitance:

(42) $$C_{q}=\frac{e^{2}}{h}\tau_{f}\;.$$

**Figure 8:**
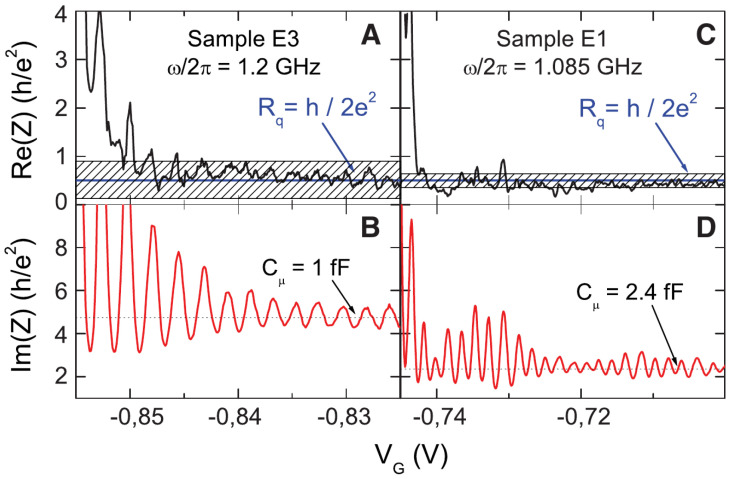
Top—Measurement of the universal charge relaxation resistance from Ref. [20]. The resistance of two different samples (E3 and E1) is given by the real part of their impedance Z=1/A as a function of the QPC potential $V_{G}$, in Figure 6, which also affected the gate potential $V_{g}$. Measurements were carried out for T=30mK and a magnetic field B=1.3T polarizing the electrons, resulting in one conducting channel. Uncertainties are indicated by the hatched areas. Bottom—Measurement of the total capacitance $C_{\mu }=C$, through Im(Z)=−1/ωC, for the same samples. The oscillatory behavior is related to resonances in the density of states of the dot.

On the experimental side, the level spacing of the quantum dot can be actually estimated and, in the experimental conditions of Ref. [20], it was established to be of the order of Δ∼15GHz, corresponding to a quantum capacitance $C_{q}∼1\;\mathrm{fF}$. Experimental measurements of *C*, reported in Figure 8, give an estimate also for $C_{g}$, showing that $C_{q}\ll C_{g}$. This implies that the level spacing Δ was much larger than the charging energy $E_{c}=e^{2}/2C_{g}$, of the order of fractions of the GHz, apparently justifying the mean-field approach to describe experimental results, with the limitations that we are going to discuss in out-of-equilibrium regimes, see Section 5.

The argument leading to Equation (41) implicitly assumes the perfect transparency of the QPC, namely that the probability amplitude *r* for a lead electron to be reflected when passing though the QPC to enter the cavity is equal to zero (r=0). In this limit, the density of states on the dot is uniform. Finite reflection r≠0 is responsible for resonant tunneling processes, leading to oscillatory behavior of the local density (or the dwell-time) of states as a function of the gate potential $V_{g}$, in agreement with the experimental findings reported in Figure 8. In Appendix B, we provide a quantitative analysis of this effect by explicitly calculating the differential capacitance $C_{0}$, Equation (39), by neglecting the term proportional to $E_{c}N^{2}$ in Equation (33).

### 4.3. The Physical Origin of the Universal Charge Relaxation Resistance

The universality of $R_{q}$ was also verified experimentally in Ref. [20], see Figure 8. In the quantum coherent regime the charge relaxation resistance is *universal*: It does not depend on the microscopic details of the circuit ($C_{g},\;r,\ldots$), but only on fundamental constants, namely Planck’s constant *h* and the electron charge *e*. This is surprising. When applying a DC voltage across a QPC connecting two leads, the QPC behaves as a resistive element of resistance [160,161]:

(43) $$R_{\mathrm{DC}}=\frac{h}{e^{2}D}\;,$$

where $D=1-{\left|r\right|}^{2}$ is the QPC transparency. This quantity depends on the amplitude *r* for electrons to be backscattered when arriving at the QPC, which can be tuned by acting on a gate potential. As we considered spinless electrons, the factor 2 in $R_{q}=h/2e^{2}$ cannot be related to spin degeneracy and D=1 in Equation (43). It is rather related to the fact that the dot is connected to a single reservoir, in contrast to the source-drain reservoirs present in DC transport experiments [56,162]. In direct transport, each metallic contact is responsible for a quantized contact resistance $R_{c}=h/2e^{2}$, the Sharvin–Imry resistance [163,164]. In source-drain experiments, Equation (43) could be recast in the form $R_{\mathrm{QPC}}+2R_{c}$, with $R_{\mathrm{QPC}}=\frac{h}{e^{2}}\frac{1-D}{D}$ the resistive contribution proper to the QPC. For the case of a mesoscopic capacitor, there is a single reservoir and one would thus expect for the charge relaxation resistance:

(44) $$R_{q}^{\mathrm{expected}}=R_{\mathrm{QPC}}+R_{c}=\frac{h}{2e^{2}}+\frac{h}{e^{2}}\frac{1-D}{D}\;.$$

The fact that $R_{q}$ does not depend on the transparency *D*, assuming the universal value $h/2e^{2}$, cannot be attributed to the laws governing DC quantum transport, and this is the reason why one can speak about the *Violation of Kirchhoff’s Laws for a Coherent RC Circuit* [20]. The universality of $R_{q}$ is rather a consequence of the fact that $R_{q}$, differently from the contact resistance $R_{c}$, is related to energy (Joule) dissipation. As electrons propagate coherently within the cavity, they cannot dissipate energy inside it, but only once they reach the lead. We will illustrate in Section 4.5 how this phenomenon is a direct consequence of the possibility to excite particle-hole pairs by driven electrons. At low frequency, dissipation is thus only possible in the presence of a continuum spectrum, accessible in the metallic reservoirs. The expected resistance (44) is recovered only if electrons lose their phase coherence inside the dot [56,162], see also Refs. [165,166], which take Coulomb blockade effects into account. For instance, in the high temperature limit $k_{B}T\gg \Delta$, Equation (44) is not recovered. The reason is that, in scattering theory, temperature is fixed by the reservoirs without affecting the coherent/phase-preserving propagation in the mesoscopic capacitor.

### 4.4. The Open-Dot Limit

We address now the role of the charging energy $E_{c}$ and come back to our initial example of the open dot limit, considered in Section 2. The possibility to rely on an exact bosonized solution for the model (1)–(2), made possible the derivation of the admittance A(ω) in linear-response theory for a fully transparent point contact (r=0) and a finite-sized cavity [65]:

(45) $$A\left(\omega \right)=-i\omega C_{g}{\left(1-\frac{i\omega \tau_{c}}{1-e^{i\omega \tau_{f}}}\right)}^{-1}\;.$$

This expression is important as it makes possible to study the interplay between two different time-scales, namely the time of flight $\tau_{f}$ of electrons inside the cavity, already present in the previous discussion, and $\tau_{c}=hC_{g}/e^{2}$ the time scale corresponding to the charging energy $E_{c}$. We mention that, interestingly, the admittance (45) was also found to describe the coherent transmission of electrons through interacting Mach–Zehnder interferometers [167,168].

What is quite remarkable about the admittance (45) is that, to linear order in ω, the two time scales $\tau_{f}$ and $\tau_{c}$ still combine into the universal charge relaxation resistance $R_{q}=h/2e^{2}$ and a series of a geometrical and quantum capacitance $C_{q}=e^{2}\tau_{f}/h$ [51,52,53] (see also Equation (42)):

(46) $$\frac{1}{C_{0}}=\left[\frac{1}{C_{g}}+\frac{h}{e^{2}\tau_{f}}\right]\;.$$

The low-frequency behavior of Equation (45) illustrates how interacting systems behave as if interactions were absent at low energies. What is then also implicit in Equation (45) is that, to observe separate effects on the charge dynamics, induced by free propagation ($\tau_{f}$) or interactions ($\tau_{c}$), one has to consider proper out-of-equilibrium/high-frequency regimes. These regimes will be addressed in Section 5.

Nevertheless, interactions still matter even in low-frequency regimes. Consider the infinite-size (metallic) limit for the cavity, $\tau_{f}\to \infty$. In this limit, also describing the experiment in Figure 2, one implicitly assumes that the driving frequency ω is larger than the internal level spacing of the dot Δ,

(47) ℏω≫Δ.

**Figure 9:**
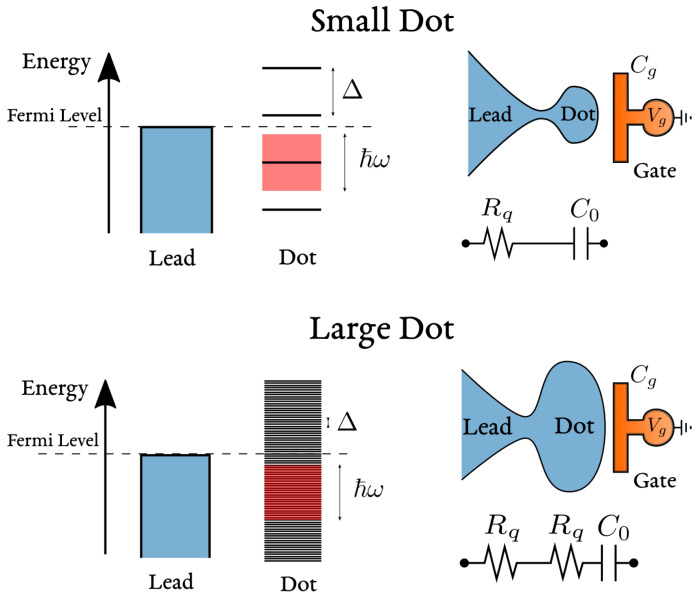
Mesoscopic crossover in the charge relaxation resistance. Top—In a small dot, the level spacing Δ is larger than the driving energy ℏω and energy levels in the dot are not excited. The universal resistance $R_{q}=h/2e^{2}$ of the equivalent *RC* circuit is furnished exclusively by the lead electron reservoir. Bottom—Excitation of energy levels inside the dot are permitted in the large dot limit, which acts as a further dissipative reservoir in series to the lead.

The discrete spectrum of the dot can thus be treated as a continuum, which allows for energy dissipation also inside the cavity, see Figure 9. In particular, averaging the admittance (45) over a finite bandwidth δω, such that ω≫δω≫Δ, one exactly recovers the admittance of a classical RC circuit of capacitance $C_{g}$ and charge-relaxation resistance $R_{q}=h/e^{2}$ [65]:

(48) $$A\left(\omega \right)=\frac{-i\omega C_{g}}{1-i\omega C_{g}\frac{h}{e^{2}}}\;.$$

The mesoscopic crossover $R_{q}=h/2e^{2}\to h/e^{2}$ is an exquisite coherent effect triggered by interactions. This phenomenon has fundamentally the same origin of the elastic electron transfer exemplified by the correlation function (3), considered at the very beginning of this review.

Remarkably, the universality of the charge-relaxation resistance holds in the presence of backscattering at the dot entrance, without affecting the mesoscopic crossover $R_{q}=h/2e^{2}\to h/e^{2}$ [65]. Nevertheless, the possibility to interpret the differential capacitance as a series of two separate geometric and quantum term as in Equation (46), is lost. If we locate the entrance of the dot at x=0, backscattering corrections to the model (1)–(2) read:

(49) $$H_{r}=-\hbar rv_{F}\left[\Psi_{R}{\left(0\right)}^{†}\Psi_{L}\left(0\right)+\Psi_{L}{\left(0\right)}^{†}\Psi_{R}\left(0\right)\right]\;,$$

and compromise a non-interacting formulation of the problem, even in its bosonized form [65,72,148].

It becomes then important to understand why and to which extent quantities such as the charge-relaxation resistance show universal coherent behavior even in the presence of interactions. The extension of the LFL theory in the quasistatic approximation provides the unified framework to understand the generality of such phenomena.

### 4.5. The Tunneling Limit and the Quasi-Static Approximation

In Section 3, we showed that a large class of models of the form (4) are effectively described, in the low-energy limit, by a LFL theory (8), in which the potential scattering coupling constant *W* depends on the orbital energy of the dot $\varepsilon_{d}$. The expansion of the charging energy Hamiltonian (33) made apparent that this energy is renormalized by the gate potential $\varepsilon_{d}\to \varepsilon_{d}-eV_{g}\left(t\right)$. For an AC bias voltage, we consider then a periodic function of time oscillating at the frequency ω:

(50) $$\varepsilon_{d}\left(t\right)=\varepsilon_{d}^{0}+\varepsilon_{\omega }\cos\left(\omega t\right)\;.$$

The *quasi-static approximation* consists in substituting Equation (50) directly in Equation (8). This condition assumes that the low energy Hamiltonian (8), derived for the equilibrium problem, follows, without any delay, the orbital oscillations expected from the parent, high-energy, model. The quasi-static approximation is then a statement about a behavior close to adiabaticity.

We consider then the linear response regime and expand the coupling $W(\varepsilon_{d})$ in $\varepsilon_{\omega }$. Focusing on the single channel case, the extension to multiple channels being straightforward, Equation (8) becomes:

(51) $$H=\sum_{k}\varepsilon_{k}c_{k}^{†}c_{k}+\left[W\left(\varepsilon_{d}^{0}\right)+W^{\prime }\left(\varepsilon_{d}^{0}\right)\varepsilon_{\omega }\cos\left(\omega t\right)\right]\sum_{kk^{\prime }}c_{k}^{†}c_{k}^{\prime }\;.$$

We diagonalize the time independent part of this Hamiltonian [104]:

(52) $$H=\sum_{kk^{\prime }}\varepsilon_{k}a_{k}^{†}a_{k}+\frac{W^{\prime }\left(\varepsilon_{d}^{0}\right)}{1+{\left[\pi \nu_{0}W\left(\varepsilon_{d}^{0}\right)\right]}^{2}}\varepsilon_{\omega }\cos\left(\omega t\right)\sum_{kk^{\prime }}a_{k}^{†}a_{k^{\prime }}\;,$$

where the operators *a* and $a^{†}$ describe the new quasi-particles diagonalizing the time independent part of the Hamiltonian (51). The Friedel sum rule (11) establishes that:

(53) $$\chi_{c}=\frac{\nu_{0}W^{\prime }\left(\varepsilon_{d}^{0}\right)}{1+{\left[\pi \nu_{0}W\left(\varepsilon_{d}^{0}\right)\right]}^{2}}\;,$$

and the Hamiltonian (52) can be cast in the more compact and transparent form:

(54) $$H=\sum_{kk^{\prime }}\varepsilon_{k}a_{k}^{†}a_{k}+\frac{\chi_{c}}{\nu_{0}}\varepsilon_{\omega }\cos\left(\omega t\right)\sum_{kk^{\prime }}a_{k}^{†}a_{k^{\prime }}\;.$$

This Hamiltonian shows the mechanism responsible for energy dissipation at low energy for the rich variety of strongly interacting systems satisfying the Friedel sum rule and LFL behavior at low energy. The time dependent term pumps energy in the system, which is then dissipated by the creation of particle-hole pairs. Crucially, this term is controlled by the static charge susceptibility $\chi_{c}$ of the quantum dot. The non-interacting Hamiltonian (54) explains why non-interacting results also hold for the universal charge relaxation resistance in the presence of interactions on the quantum dot.

We now illustrate how the Hamiltonian (54) implies the validity of the KS relation and thus universality of the charge-relaxation resistance $R_{q}$. The proof was originally devised for spin-fluctuations [169] and we extend it here to the case of charge fluctuations. For drives of the form (50), the power dissipated by the system is proportional to the imaginary part of the dynamic charge susceptibility, see Appendix C,

(55) P=12εω2ωImχc(ω).

A direct calculation of Imχc is a difficult task and this is where the low-energy model (54) becomes useful. Similarly as for Equation (55), the LFL theory (54) predicts the dissipated power:

(56) P=12εω2ωImχA(ω),

where the linear response function $\chi_{A}\left(t-t^{\prime }\right)=\frac{i}{\hbar }\theta \left(t-t^{\prime }\right){\langle \left[A\left(t\right),A\left(t^{\prime }\right)\right]\rangle }_{0}$ is a correlator at different times of the potential scattering operator:

(57) $$A=\frac{\chi_{c}}{\nu_{0}}\sum_{kk^{\prime }}a_{k}^{†}a_{k^{\prime }}\;,$$

responsible for the creation of particle-hole pairs. The Fourier transform of the response function reads:

(58) $$\chi_{A}\left(\omega \right)=-\frac{1}{\hbar }\frac{\chi_{c}^{2}}{\nu_{0}^{2}}\sum_{pp^{\prime }}f\left(\varepsilon_{p}\right)\left[1-f\left(\varepsilon_{p^{\prime }}\right)\right]\left[\frac{1}{\omega +\frac{\varepsilon_{p}-\varepsilon_{p^{\prime }}}{\hbar }+i0^{+}}-\frac{1}{\omega +\frac{\varepsilon_{p^{\prime }}-\varepsilon_{p}}{\hbar }+i0^{+}}\right]\;,$$

in which $f\left(\varepsilon_{p}\right)=1/\left(e^{\beta \varepsilon_{p}}+1\right)$ is the Fermi distribution. We consider the electron lifetime as infinite, i.e., much longer than the typical time scales $\tau_{c}$ and $\tau_{f}$. Taking the imaginary part and the continuum limit for the spectrum in the wide-band approximation, one finds, at zero temperature,

(59) ImχA(ω)=πℏωχc2.

The two dissipated powers (55) and (59) have to be identical, implying the Korringa–Shiba relation (32), enforcing then a universal value for the charge relaxation resistance $R_{q}=h/2e^{2}$.

### 4.6. The LFL Theory of Large Quantum Dots: The Mesoscopic Crossover $R_{\;q}=h/2e^{2}\to h/e^{2}$

The above demonstration has to be slightly adapted to show the mesoscopic crossover $R_{q}=h/2e^{2}\to h/e^{2}$. This crossover takes place for the CBM (12), in the infinite-size limit of the dot. As implicit in the effective description (21) of the CBM, the dot and the lead constitute two separate Fermi liquids. Section 2 and Section 3 illustrated how the energy cost $E_{c}$ prevents the low-energy transfer of electrons between the dot and lead [77]. The electrons of both these gases are then only backscattered at the lead/dot boundary with opposite amplitudes. In the quasi-static approximation, all the steps carried in the previous discussion apply for the Hamiltonian (21). In this case, the time variation of the orbital energy $\varepsilon_{d}$ also drives particle-hole excitations in the dot. The operator responsible for energy dissipation becomes:

(60) $$A=\frac{\chi_{c}}{\nu_{0}}\left(\sum_{kk^{\prime }}c_{k}^{†}c_{k^{\prime }}-\sum_{ll^{\prime }}d_{l}^{†}d_{l^{\prime }}\right),$$

in which the operators $c_{k}^{†}$ and $d_{l}^{†}$ create lead and dot electrons of energy $\varepsilon_{k,l}$ respectively. This formulation of the operator *A* adds a further contribution to Equation (58), analogous to the contribution of particle-hole pairs excited in the lead, namely:

(61) $$-\frac{1}{\hbar }\frac{\chi_{c}^{2}}{\nu_{0}^{2}}\sum_{ll^{\prime }}f\left(\varepsilon_{l}\right)\left[1-f\left(\varepsilon_{l^{\prime }}\right)\right]\left[\frac{1}{\omega +\frac{\varepsilon_{l}-\varepsilon_{l^{\prime }}}{\hbar }+i0^{+}}-\frac{1}{\omega +\frac{\varepsilon_{l^{\prime }}-\varepsilon_{l}}{\hbar }+i0^{+}}\right]\;.$$

The limits ω→0 and Δ→0 do not commute in the above expression. This fact has a clear physical interpretation: If the frequency is sent to zero before the level spacing, energy cannot be dissipated in the cavity and no additional contribution to Imχc(ω) is found. If the opposite limit is taken, the condition (47) is met and the Korringa–Shiba relation is then modified by a factor two:

(62) Imχc(ω)=2πℏωχc2,

which doubles the universal value of the single-channel charge relaxation resistance $R_{q}=h/e^{2}.$ The relation (62) was originally shown by explicit perturbation theory in the tunneling amplitude, close and away from charge degeneracy points [65]. As summarized in Figure 9, driving at a frequency higher than the dot level spacing induces the creation of particle/hole pairs inside the dot as well, enhancing energy dissipation with respect to the small dot limit ℏω<Δ. As energy can be *coherently* dissipated in two fermionic baths (dot and lead), the dot acts effectively as a further (Joule) resistor in series with the lead, leading to a doubled and still universal charge relaxation resistance.

### 4.7. The Multi-Channel Case and Universal Effects Triggered by Kondo Correlations

The above discussion also extends to the *M* channels case, leading to a generalized KS relation:

(63) Imχc(ω)ω→0=ℏπω∑σχσ2,

which corresponds to a non-universal expression for the charge relaxation resistance [68,69]

(64) $$R_{q}=\frac{h}{2e^{2}}\frac{\sum_{\sigma }\chi_{\sigma }^{2}}{{\left(\sum_{\sigma }\chi_{\sigma }\right)}^{2}}\;.$$

This expression is analogous to the one obtained by Nigg and Büttiker [54]. In their derivation leading to Equation (A48), the densities of states, or dwell-times $\tau_{\sigma }$, of the σ channel in the dot, replace the susceptibilities $\chi_{\sigma }$. The single channel case is remarkable in that the numerator simplifies with the denominator in Equation (64), leading to the universal value $h/(2e^{2})$, which is thus physically robust. Otherwise, in the fine-tuned case that all the channel susceptibilities are equal, one finds $R_{q}=h/2e^{2}M$.

Spinful systems in the presence of a magnetic field are the simplest ones to study how the charge-relaxation resistance is affected by breaking the symmetry between different conduction channels. Indeed, lifting the orbital level degeneracy by a magnetic field breaks the channel symmetry and the charge relaxation resistance is no longer universal, as it was originally realized in studies of the AIM (13) relying on the Hartree–Fock approximation [54].

**Figure 10:**
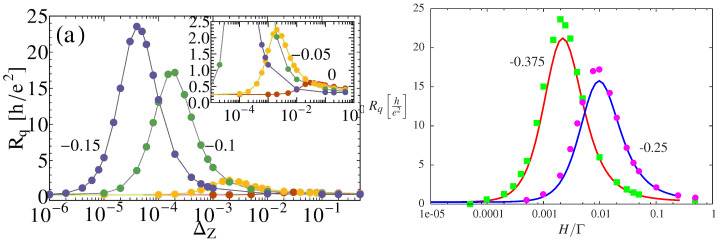
Left—Dependence of $R_{q}$ on the Zeeman splitting $\Delta_{Z}$ in the Kondo regime from Ref. [147]. These results have been obtained by Numerical Renormalization Group (NRG) calculations with Γ=0.02 and $E_{c}=0.2$ (both quantities are measured in units of the contact bandwidth *D* and the definition of the hybridization energy Γ is provided in Appendix A.3). They show that, for Zeeman energies of the order of the Kondo temperature, a giant non-universal peak appears in the charge relaxation resistance. Right—Comparison of $R_{q}$ as a function of the magnetic field between NRG calculations (dots) (extracted from Ref. [147]) and our Bethe ansatz results (solid lines) for different $\varepsilon_{d}/U$ and U/Γ=20, showing excellent agreement [67,68].

Nevertheless, the self-consistent approach misses important and sizable effects triggered by strong Kondo correlations. These were originally observed relying on the Numerical Renormalization Group (NRG) [147]. The numerical results, reported in Figure 10, showed that, for Zeeman splittings of the order of the Kondo temperature $T_{K}$, the charge relaxation resistance can reach up to 100 times the universal value of $R_{q}=h/\left(4e^{2}\right)$, which would be expected in the two-fold spin degenerate case.

The LFL approach allows the analytical quantification and physical interpretation of such giant dissipative phenomenon [67,68]. For two spin channels, the total charge on the dot is the sum of the two spin occupation $\langle N\rangle =\langle N_{↑}\rangle +\langle N_{↓}\rangle$. Equation (64) can then be recast in the useful form:

(65) $$R_{q}=\frac{h}{4e^{2}}\left(1+\frac{\chi_{m}^{2}}{\chi_{c}^{2}}\right)\;,$$

in which we introduce the usual charge susceptibility: $\chi_{c}=-\partial \langle N\rangle /\partial \varepsilon_{d}$ and the *charge-magneto* susceptibility

(66) $$\chi_{m}=-2\frac{\partial \langle m\rangle }{\partial \varepsilon_{d}}\;.$$

**Figure 11:**
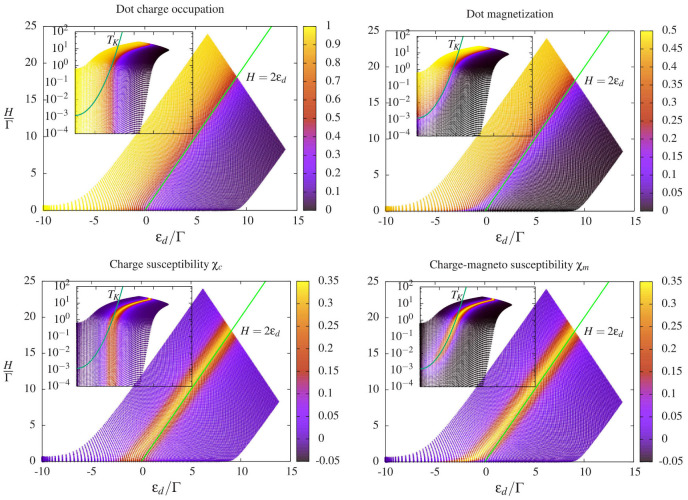
From Ref. [68]. Top—Charge occupation and magnetization of the dot for U/Γ=20 as function of the orbital energy $\varepsilon_{d}$ and magnetic field *H*. The insets show the same quantities on a logarithmic scale. The light green lines in the linear plots correspond to $H=2\varepsilon_{d}$, and separate regions with different charge occupations, while the dark green lines in the insets correspond to $T_{K}$, Equation (26), and separate regions with different magnetization. The charge is not sensitive to the formation of the Kondo singlet for Zeeman energies below the Kondo temperature (green line), while the magnetization becomes zero. Bottom—Corresponding charge susceptibility and charge-magneto susceptibility. The susceptibilities are in units of 1/Γ. In the insets the same quantities are plotted on a logarithmic scale and the zone of appearance of the giant peak of the charge relaxation resistance can be appreciated. It is the region, following $T_{K}$, in which $\chi_{c}$ is close to zero, while $\chi_{m}$ acquires important values because of the formation of the Kondo singlet.

This quantity is twice the derivative of the dot magnetization $\langle m\rangle =\left(\langle N_{↑}\rangle -\langle N_{↓}\rangle \right)/2$, with respect to the orbital energy $\varepsilon_{d}$. The charge-magneto susceptibility is an atypical object to study quantum dot systems, where the *magnetic* susceptibility $\chi_{H}=-\partial \langle m\rangle /\partial H$ is rather considered to study the sensitivity of the local moment of the quantum dot to variations of the magnetic field *H*. Equation (65) shows that the susceptibility of the *magnetization* of the dot, and not its charge, is responsible for the departure from the universal quantization $h/(4e^{2})$ of the charge relaxation resistance. Equation (65) also separates explicitly charge and spin degrees of freedom of the electrons in the quantum dot. They can display very different behaviors in correlated systems, as illustrated in Figure 11 in the Kondo regime, defined for one charge blocked on the dot and Zeeman energies below the Kondo temperature (26).

In particular, Kondo correlations strongly affect the dot magnetization, but not its occupation. The points where $\chi_{m}$ differs from $\chi_{c}$ correspond to non-universal charge relaxation resistances. In Figure 10, the values derived with the LFL approach (65) are compared to those obtained with NRG [147], showing excellent agreement. Additionally, the LFL approach also allows to derive an exact analytical description of this peak, showing a genuinely giant dissipation regime: Simultaneous breaking of the SU(2) (H≠0) and particle-hole symmetry ($\varepsilon_{d}\neq -U/2$) trigger a peak in $R_{q}$ which scales as the 4th(!) power of U/Γ and has its maximum for Zeeman splittings of the order of the Kondo temperature. This effect is caused by the fact that breaking the Kondo singlet by a magnetic field activates spin-flip processes, which dissipate energy through creation of particle-hole pairs [147].

**Figure 12:**
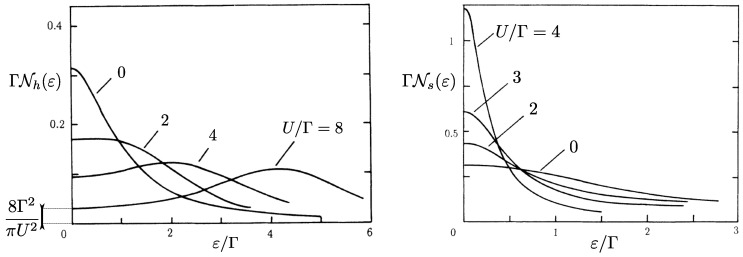
From Ref. [170]. (**Left**)—Density of states of local holons on the dot $N_{h}\left(\varepsilon \right)$. ε is the excitation energy. A Coulomb peak emerges increasing the interaction parameter U/Γ and vanishes at zero energy as $8\Gamma /\pi U^{2}$. This quantity coincides with $\chi_{c}$, plotted in Figure 11. (**Right**)—Density of states of local spinons $N_{s}\left(\varepsilon \right)$. It behaves as the holonic one for U/Γ=0 and develops the Abrikosov–Suhl resonance at zero energy, the signature of the formation of the strongly correlated Kondo singlet state.

We conclude by discussing the deviations of the differential capacitance $C_{0}$ from the local density of states of the cavity, which is clearly apparent in Kondo regimes. The spin/charge separation arising in the AIM allows to observe important physical effects on the differential capacitance of strongly interacting systems. Charge and spin on the dot are carried by different excitations: Holons and spinons. We report in Figure 12 the density of states of these excitations in the particle-hole symmetric case $\varepsilon_{d}=-U/2$. In the absence of interactions (U/Γ=0), they have the same shape, but they start to strongly differ as the interaction parameter U/Γ is increased. They develop well pronounced peaks, but at different energies, signaling the appearance of separated charge and spin states. In the case of holons, the excited charge state appears close to ε=U/2, the energy required to change the dot occupation at particle-hole symmetry. In Ref. [170], it is shown that the density of states of the holons equals the static charge susceptibility $\chi_{c}$, coinciding then with the differential capacitance $C_{0}$. At particle-hole symmetry, this quantity scales to zero as $8\Gamma /\pi U^{2}$, see Equation (29). Instead, the spinon density of states develops a sharp peak at zero energy, known as the Abrikosov–Suhl resonance [118], signaling the emergence of the strongly correlated Kondo singlet. The differential capacitance $C_{0}$ is completely insensitive to this resonance, which dominates the *total* density of states on the dot. Such an effect was distinctly observed in carbon nanotube devices coupled to high-quality-factor microwave cavities [145]. These systems efficiently probe the admittance (30) also in quantum dots with more than two internal degrees of freedom [150,152], such as extensions of the AIM to SU(4) regimes, relevant for quantum dots realized with carbon nanotubes [68,171,172,173,174,175,176,177].

The above discussion completes the review of the application of the LFL theory to study the low-energy dynamics of quantum impurity driven systems. Further applications could be envisioned to describe various correlation effects on different aspects of weakly driven interacting quantum-dot systems, as long as they can be described by an effective theory of the form (8). An important case involves the driving of the coupling term $H_{\mathrm{res}-\mathrm{dot}}\to H_{\mathrm{res}-\mathrm{dot}}\left(t\right)$ in the Hamiltonian (4), which has been implemented experimentally, with important metrologic applications [27,28,29,30,31,32]. Another interesting perspective concerns the application of the LFL theory to energy transfer [178,179,180], or coupling quantum-dot systems to mechanical degrees of freedom [181,182,183,184,185,186,187], which are described by similar models as quantum-dot devices embedded in circuit-QED devices [150,153,154,155,156,157,158].

Deviations from universal and coherent behaviors are expected in non-LFL regimes, arising when the reservoirs are Luttinger Liquids [188,189] or in over-screened Kondo impurities, in which the internal degrees of freedom of the bath surpass those of the impurity [85,190].

We have thus illustrated how a coherent and effectively non-interacting LFL theory accounts for strong correlation effects in the dynamics of quantum dot devices. It has to be clarified how interaction are supposed to affect proper out-of-equilibrium regimes. As a direct example, consider again the admittance (45). Its expansion to low-frequencies completely reproduces the self-consistent predictions of Refs. [51,52,53], but it ‘hides’ the qualitative difference between the two time scales $\tau_{c}$ and $\tau_{f}$, associated to interactions and free-coherent propagation respectively. Higher-frequency driving will inevitably unveil this important difference, as we are going to demonstrate by giving a new twist to past experimental data in the next conclusive section.

## 5. What about Out-Of-Equilibrium Regimes? A New Twist on Experiments

We conclude this review by showing how interaction inevitably dominate proper out-of-equilibrium or fastly-driven regimes. We will focus, also in this case, on the mesoscopic capacitor. In particular, we will show that past experimental measurements, showing fractionalization effects in out-of-equilibrium charge emission from a driven mesoscopic capacitor [25], also manifest previously overlooked signatures of non-trivial many-body dynamics induced by interactions in the cavity.

As a preliminary remark, notice that the circuit analogy (31) does not apply for a non-linear response to a gate voltage change or to fast (high-frequency) drives. An important example is a large step-like change in the gate voltage $V_{g}\left(t\right)=V_{g}\theta \left(t\right)$, θ(t) being the Heaviside step function, which is relevant to achieve triggered emission of quantized charge [24]. Such a non-linear high-frequency response has been considered extensively for non-interacting cavities [22,24,191,192,193,194,195], where the current response to a gate voltage step at time t=0 was found to be of the form of simple exponential relaxation [22,191,193,195]:

(67) $$I\left(t\right)\propto e^{-t/\tau_{R}}\theta \left(t\right).$$

For a cavity in the quantum Hall regime the relaxation time $\tau_{R}=\tau_{f}{/(1-|r|}^{2})$, where $\tau_{f}$ is the time of flight around the edge state of the cavity, see Figure 6 and Figure 7, and *r* the reflection amplitude of the point contact.

There have been relatively few studies of the out-of-equilibrium behavior of the mesoscopic capacitor in the presence of interactions. The charging energy leads to an additional time scale $\tau_{c}=2\pi \hbar C_{g}/e^{2}$ for charge relaxation. The limit $1-{\left|r\right|}^{2}\ll 1$ of a cavity weakly coupled to the lead, such that it can effectively be described by a single level, was addressed in Refs. [196,197,198,199,200,201].

The full characterization of the out-of-equilibrium dynamics behavior of the mesoscopic capacitor, with a close-to-transparent point contact, was carried out in Ref. [148], extending the analysis of Ref. [65] to a non-linear response in the gate voltage $V_{g}$. A main result, spectacular in its simplicity, is that for a fully transparent contact (r=0) the linear-response admittance (45) also describes the non-linear response, i.e., the correction terms in Equation (30) vanish for an ideal point contact connecting cavity and lead [136,202,203,204]. The Fourier transform of the admittance (45) describes the real-time evolution of the charge Q(t) after a step change in the gate voltage.

**Figure 13:**
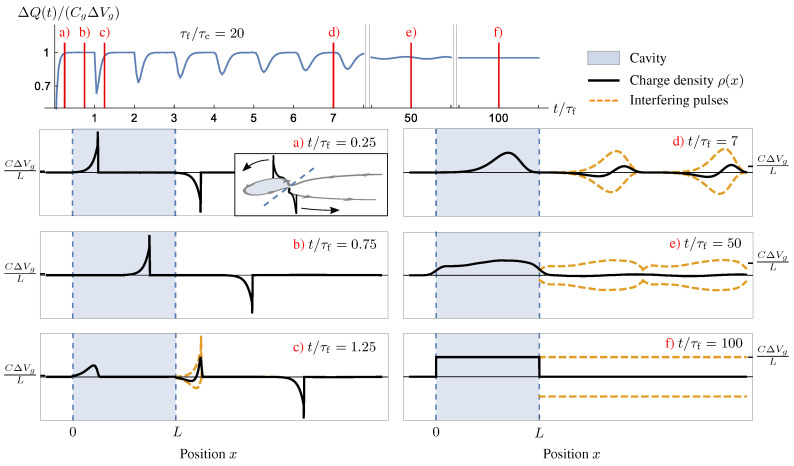
From Ref. [148]. Time-evolution of the current/charge density following a sudden gate voltage shift at time t=0 for large interaction strength, $\tau_{f}=20\tau_{c}$. Top—Charge response ΔQ(t) as a function of time *t*. Bottom—Series of snapshots of the current/charge density j(x,t) at different times. In the inset of panel (**a**), the real-space representation (reproducing the one adopted in Figure 7 with the dot site ℓ=L) of the mesoscopic capacitor with the profiles of the emitted charge pulses is given. The times at which the snapshots are taken are indicated by vertical dashed lines in the top panel. Notice that the scale changes along the vertical axis in the different panels. At time t=0, two charge pulses of width $∼v_{F}\tau_{c}$ and opposite sign emerge from the point contact (**a**,**b**), one pulse entering the cavity and one pulse entering the chiral edge of the bulk two-dimensional electron gas. Both pulses have a net charge approaching $C_{g}\Delta V_{g}$. The pulse that is emitted into the cavity returns to the point contact at time $t=\tau_{f}$. As that pulse leaves the cavity, a second pulse-antipulse pair is generated (**c**), partially canceling the original charge pulse that leaves the cavity at $t=\tau_{f}$. The resulting pulse exiting the cavity is the sum of the dashed profiles. The repetition of this mechanism leads to the widening and lowering of successive pulses (**d**,**e**) (notice the change of scale between snapshots). Finally, the asymptotic configuration is attained with a charge $C\Delta V_{g}$ uniformly distributed along the cavity edge (**f**).

Figure 13 illustrates that initially, for times up to $\tau_{f}$, Q(t) relaxes exponentially with time $\tau_{c}$, whereas at time $t=\tau_{f}$ the capacitor abruptly enters a regime of exponentially damped oscillations, the period and the exponential decay, controlled by a complex function of $\tau_{f}$ and $\tau_{c}$, which does not correspond to any time scale extracted from low-frequency circuit analogies. This behavior is not captured by Equation (67), derived in the non-interacting limit. These oscillations correspond to the emission of initially sharp charge density pulses, which are damped and become increasingly wider after every charge oscillation. Such complex dynamics is exquisitely coherent, but totally governed by interactions.

Additionally, it is also interesting to consider the effect of a small reflection amplitude *r* in the point contact. In this case, the charge $Q_{r}$ acquires nonlinear terms in the gate voltage $V_{g}$,

(68) $$Q_{r}\left(t\right)=Q\left(t\right)-\frac{e\tilde{r}}{\pi C}\int dt^{\prime }\;A\left(t-t^{\prime }\right)\sin\left[2\pi Q\left(t^{\prime }\right)/e\right],$$

in which A(t) and Q(t) are the Fourier transform of the admittance and charge for the case of a point contact with perfect transparency, r=0, see Equations (30) and (45). The parameter $\tilde{r}$ involves both the (weak) backscattering amplitude *r* and temperature *T*, details can be found in Ref. [148].

### Experimental Signatures of the Effects of Interaction in Quantum Cavities Driven out of Equilibrium

**Figure 14:**
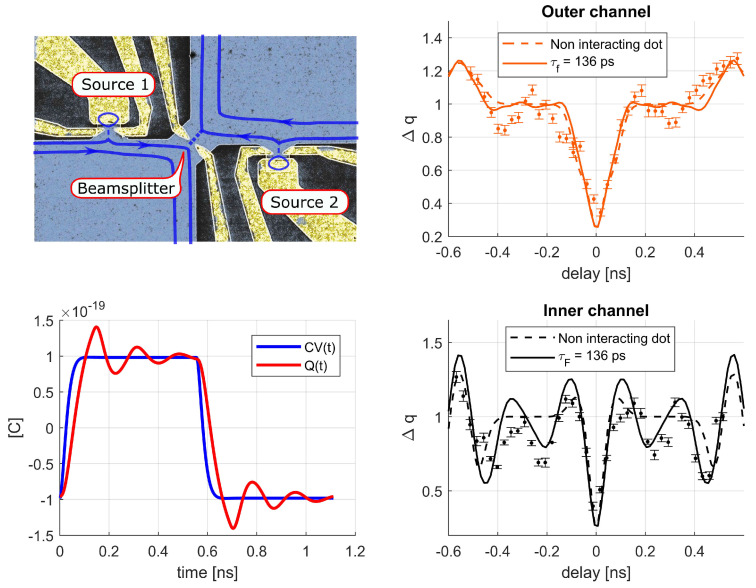
Top-Left—Hong-Ou-Mandel experiment from Ref. [25]: Two single electron sources, as that shown in Figure 6, inject single electron towards the same QPC, which works as a beamsplitter. Bottom-left—Simulation based on Ref. [148] of the charge exiting the open dot when applying a square pulse sequence V(t) with a rise time of 30 ps, one clearly sees the additional pulses coming from interactions. Top-Right—Normalized Hong-Ou-Mandel current noise Δq of the outer edge as a function of the time delay τ with which charge arrives on the QPC from different sources. Noise is suppressed for τ=0 because of anti-bunching effects, but additional oscillations were observed for τ≠0, which the theory in Ref. [148] contributed to explain. The points are the experimental data while the solid and dashed lines are theoretical curves with different fittings for the time of flight $\tau_{f}$ for electrons in the cavity. Bottom-Right—Same as in the central panel but for the inner edge.

The prediction that, in the open dot limit, interactions trigger the emission of a series of subsequent charge density pulses led to the possible explanation of additional effects that relate to a, so far not satisfactorily explained, part of the Hong-Ou-Mandel current noise measurements at the LPA [25,205]. The experimental setup is the solid state realization of the Hong-Ou-Mandel experiment, see the left panel in Figure 14: When two electrons collided at the same time on the QPC from different sources (states 1 and 2), they could not occupy the same state because of Pauli’s exclusion principle and their probability to end up in different leads (states 3 and 4) was increased. As a consequence, the current noise was suppressed [132], see the right panels in Figure 14. More generally, Δq(τ) measures the cross-correlation (or overlap) in time of the two incoming currents at the level of the QPC. If the two incoming currents are identical in each input, one should get Δq(τ=0)=0 and the rest of the curve will reflect on the time trace of the current. However, because of small asymmetries in the two electronic paths and the two electron sources, the noise suppression is not perfect [205]. In Ref. [25], the current noise Δq as a function of the time delay τ with which electrons arrived at the QPC from different sources was measured in more detail for the outer and inner edge of the filling factor ν=2 (central and right panel in Figure 14). The current in the inner edge channel was induced by inter-edge Coulomb interactions and could be computed with a plasmon-scattering formalism [25,206]. In addition to what this plasmon scattering model predicted, unexpected oscillations as a function of τ were observed. These could be satisfactorily explained by our prediction [148] of further charge emission triggered by interactions in the electron sources [205]. From independent calibration measurements, the total RC time constant of the source could be measured to set the constrain ${\left(\tau_{f}^{-1}+\tau_{c}^{-1}\right)}^{-1}/2=\tau_{RC}=21$ ps. Combining Equation (30) and (45) with the plasmon scattering formalism one could compute the current noise in the ouput of the Hong-Ou-Mandel interferometer Δq(τ) with only one fitting parameter: The ratio $\tau_{f}/\tau_{c}$. The minimization procedure gave the most-likely result: $\tau_{f}=136$ ps. The comparison is shown on the right panels of Figure 14, where the model for $\tau_{f}=2\tau_{RC}$ [$\tau_{c}\to \infty$, i.e., no interaction within the dot] described the fractionalization process due to interedge Coulomb interactions but not the interactions within the dot itself. This provides a reasonable qualitative and quantitative agreement with the experimental data reported in Ref. [25]. On the bottom-left panel in Figure 14 we compared, for $\tau_{f}=136$ ps, the charge exiting the dot Q(t) with the applied square pulse sequence on the top-gate V(t) which had a finite rise time of 30 ps. In particular, it could explain the appearance of extra rebounds in Δq for time delays τ between 70 and 450 ps which was not possible with a non-interacting dot ($\tau_{f}=0$). This was directly due to the additional effects coming from the interactions within the dot itself and could not be explained by the fractionalization mechanism. Indeed, relying exclusively on the model describing fractionalization, we could not reproduce the pronounced additional rebound for |τ|=200 ps for $\tau_{f}<100$ ps. This highlights the relevance of Coulomb interactions in the open dot dynamics.

## 6. Conclusions

This review addressed the importance of interactions for the investigation and control of dynamical quantum coherent phenomena in mesoscopic quantum-dot devices.

In Section 2, we discussed how interactions are the essential ingredient allowing long-range quantum state transfer in mesoscopic devices. Notice that the same phenomenon has been suggested to enforce nonlocal phase-coherent electron transfer in wires supporting topologically protected Majorana modes at their edges [207,208]. Such an effect is currently being considered in various Majorana network models for stabilizer measurements in corresponding implementations of topological quantum error correction codes [209,210,211].

The local Fermi liquid approach, discussed in Section 3, provides the unifying theoretical framework to describe the low-energy dynamics of such various mesoscopic devices. Its application to the various experimental setups mentioned in the Introduction, will be definitively useful to bring further understanding in the complex and rich field of out-of-equilibrium many-body systems. The insight given on universal quantum dissipation phenomena, discussed for the mesoscopic capacitor in Section 4, and, in particular, the novel interaction effects, unveiled in the experiment discussed in Section 5, give two clear examples of the utility of this approach.

Beyond the already mentioned potential for quantum dot devices coupled to microwave cavities [150,151,152,153,154,155,156,157,158,159] and to energy transfer [178,179,180], important extensions of the LFL approach should be envisioned for understanding the properties of mesoscopic devices involving non-Fermi liquids at the place of normal metallic leads. The most important cases would involve superconductors [18,19,212,213,214,215,216,217] or fractional Quantum Hall edges states, in which quantum noise measurements have been crucial to address and unveil the dynamics of fractionally charged excitations [218,219,220,221,222,223,224,225,226,227,228,229,230,231,232,233,234,235]. Additionally, the recent realization of noiseless levitons [8,9,10,11,12] paves the way to interesting perspectives to investigate flying anyons [13,220,236] and novel interesting dynamical effects [188,189,237].

## Figures and Tables

**Figure A1:**
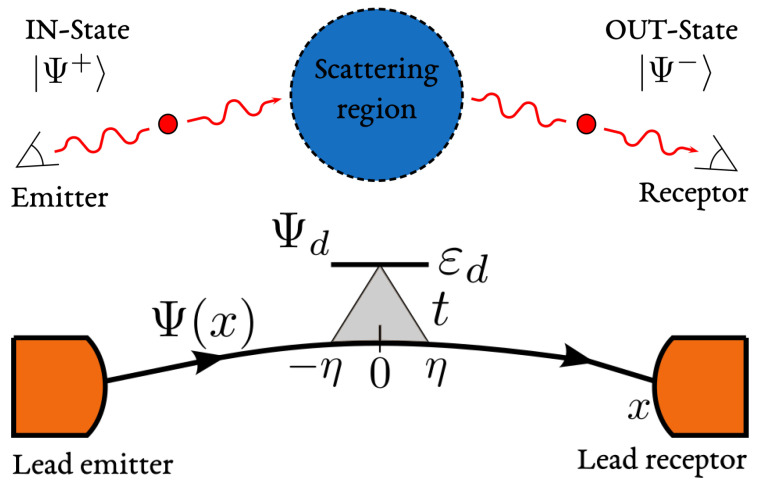
Top—Illustration of the physical situation described by the scattering formalism. Electron wave packets are emitted in the IN-State $|\Psi^{+}\rangle$ and then measured in the OUT-state $|\Psi^{-}\rangle$ once they have passed through the scattering region. Bottom—Realization of the scattering setup with a quantum Hall chiral edge state tunnel coupled (in a region of size 2η) with a resonant level of energy $\varepsilon_{d}$.

**Figure A2:**
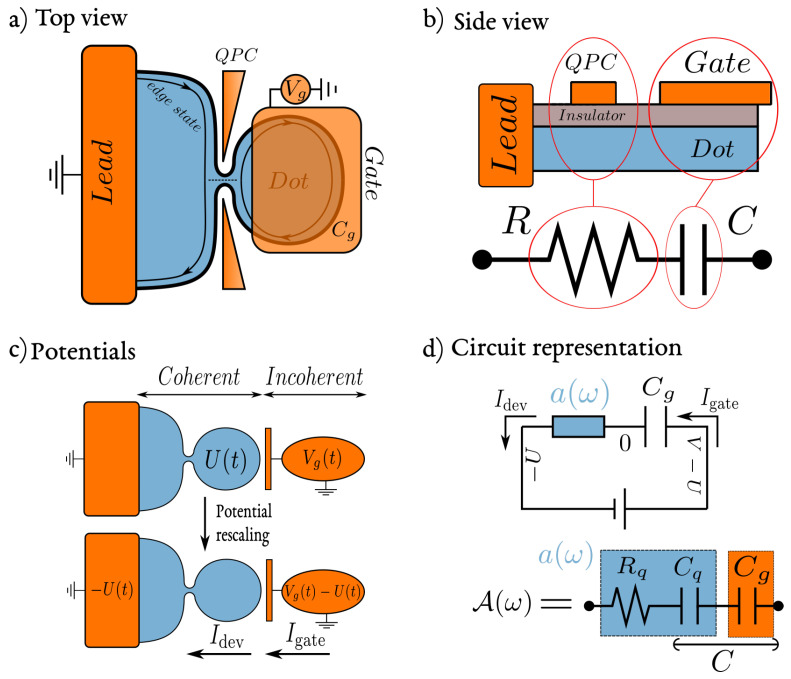
(**a**,**b**) Schematic representation of the quantum *RC* circuit. Electrons in the edge states of a two-dimensional electron gas (2DEG) in the integer quantum Hall regime tunnel inside a quantum dot through a QPC. The dot is driven by a top metallic gate. The dot and the gate are separated by an insulator and cannot exchange electrons, thus forming the two plates of a capacitor *C*. The QPC is a resistive element of resistance *R*. These two circuit elements are in series and define a quantum coherent *RC* circuit. (**c**,**d**) The blue region of the two-dimensional gas is phase coherent. The top metallic gate is an incoherent metal, driven by a time-dependent gate potential $V_{g}\left(t\right)$, which induces an unknown uniform potential U(t) on the dot. The classical circuit analogy in (**d**) is made possible by shifting all energies by −U(t). The whole device behaves as a charge relaxation resistance $R_{q}$ in series to a total capacitance *C*: series of a quantum and geometrical capacitance $C_{q}$ and $C_{g}$.

**Figure A3:**
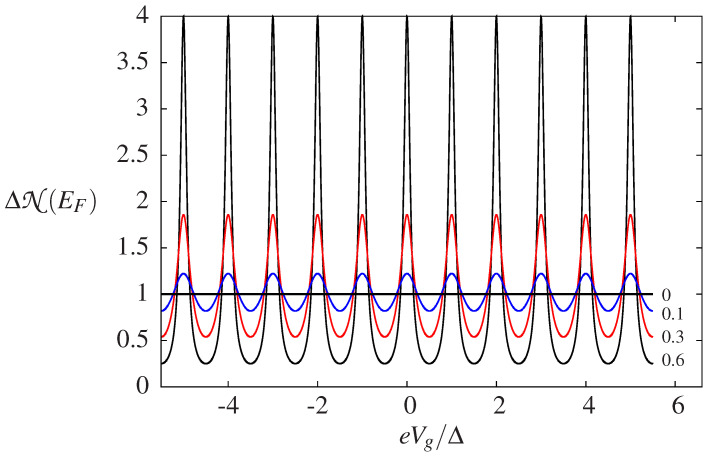
Peaked structure of the local density of states N(ε) on the dot as a function of the orbital energy shift controlled by the gate potential $eV_{g}$ from Equation (A45). $N(E_{F})$ is plotted for different values of the backscattering amplitude *r*. The progressive opening of the dot drives a transition from a Lorentzian to an oscillatory behavior of $C_{q}$, coherent with the experimental measurements illustrated in Figure 8. For a completely transparent dot (r=0) the density of states is uniform, which implies $C_{0}=e^{2}/\Delta$.

## Abbreviations

The following abbreviations are used in this manuscript:

QPC	quantum point contact
LFL	local Fermi liquid
CBM	Coulomb blockade model
AIM	Anderson impurity model
SW	Schrieffer–Wolff
DC	direct current
2DEG	two-dimensional electron gas
KS	Korringa–Shiba
AC	alternate current
NRG	numerical renormalization group

## Appendix A. Scattering Theory, Phase-Shifts and the Friedel Sum Rule

In this Appendix we review some useful results from scattering theory. In Appendix A.1, we provide the definition of the S- and T-matrix in scattering theory. In Appendix A.2, we derive the Friedel sum rule for a non-interacting electron gas with an elastic impurity. In Appendix A.3, we illustrate these concepts on the simple case of a chiral edge state tunnel coupled to a single resonant level. In Appendix A.4, we derive the phase-shift induced on lead electrons by the scattering potential of the LFL, Equation (8).

### Appendix A.1. General Definitions

We aim at describing the general situation of Figure A1, which is reproduced by mesoscopic settings, such as a single resonant level coupled to a chiral edge state, which also describes the mesoscopic capacitor in the non-interacting limit, see also Figure 7 in the main text. Consider a wave packet emitted and detected in the distant past and future, namely t=−∞ and t=+∞, and which enters a scattering region at time t=0. Close to detection and emission, it is assumed that the wave packet does not feel the presence of the scatterer, whose interaction range is delimited inside the dashed line in Figure A1. The system is described by a single-particle Hamiltonian of the form:

(A1) $$H=H_{0}+V\;,$$

in which $H_{0}$ describes the free propagation of a wave-packet and V the scatterer. The T-matrix describes the effects of a scatterer on the propagation of a free particle. It is an improper self-energy for the resolvent of the lead electrons, which appears in a modified form of Dyson’s equation:

(A2) $$\begin{array}{cccc} G(z) & =G_{0}\left(z\right)+G_{0}\left(z\right)T\left(z\right)G_{0}\left(z\right)\;, & G(z) & =\frac{1}{z-H}\;, \end{array}$$

in which z is a complex number. $G_{0}$ is the free resolvent describing free electrons:

(A3) $$G_{kk^{\prime }}^{0}\left(z\right)=\langle k|G^{0}\left(z\right)|k^{\prime }\rangle =\frac{\delta_{kk^{\prime }}}{z-\varepsilon_{k}},$$

the $|k\rangle$ states being the single particle eigenvectors of the unperturbed Hamiltonian $H_{0}$. One can readily show that the T-matrix reads:

(A4) $$T\left(z\right)=V{\left(I-G_{0}\left(z\right)V\right)}^{-1}\;.$$

The general definition of the phase-shift, a key quantity within scattering theory [238,239], reads:

(A5) $$\delta_{\varepsilon }=\arg\left[T(\varepsilon +i0^{+})\right]\;.$$

We define the IN and OUT states $|\Psi^{\pm }\rangle$ as the eigenvectors of energy ε of the Hamiltonian of the whole system, including the scattering region, as the states coinciding asymptotically with free plane waves in the past and in the future respectively. The scattering matrix S gives the overlap between these two states:

(A6) $$S_{kk^{\prime }}\left(\varepsilon \right)=\langle \Psi_{k}^{-}|\Psi_{k^{\prime }}^{+}\rangle \;,$$

where $k/k^{\prime }$ are the momenta of the OUT/IN states. The T- and S-matrix are related by the relation [238,239]:

(A7) $$S_{kk^{\prime }}=\delta_{kk^{\prime }}-2\pi i\delta \left(\varepsilon_{k}-\varepsilon_{k^{\prime }}\right)T_{kk^{\prime }}\;,$$

or equivalently, in the energy representation,

(A8) $$S\left(\varepsilon \right)=I-2\pi i\nu_{0}T\left(\varepsilon \right)\;.$$

The S-matrix is unitary and in the single channel case it is completely defined by a phase $S\left(\varepsilon \right)=e^{2i\delta_{\varepsilon }}$. The phase $\delta_{\varepsilon }$ is the phase-shift caused by scattering and in general the condition $S\left(\varepsilon \right)=e^{2i\delta_{\varepsilon }}$ is always verified if we take the definition of the phase-shift directly from the T-matrix:

(A9) $$T\left(\varepsilon \right)=-\frac{1}{\pi \nu_{0}}\sin\delta_{\varepsilon }e^{i\delta_{\varepsilon }}\;.$$

There is an interesting connection with the Kondo regime. In Section 3.3.2, we illustrated how particle-hole symmetry enforces that the phase-shift is given by $\delta =\delta_{K}=\pi /2$. The scattering matrix thus equals the identity. This case is also known as the unitary limit of the Kondo model, in which the transmission probability through a Kondo correlated dot is unity.

### Appendix A.2. The Friedel Sum Rule

We show here the Friedel sum rule for non-interacting electrons scattering on an elastic impurity. We first consider the total electron occupation $\langle N\rangle$ of an electron gas, which reads:

(A10) $$\langle N\rangle =\sum_{\alpha }\int_{-\infty }^{\infty }d\omega A_{\alpha }\left(\omega \right)f\left(\omega \right)\;,$$

the sum on the label α running over all the eigenstates of the Hamiltonian (A1). $A_{\alpha }\left(\omega \right)$ is the spectral function of the state α, defined as:

(A11) Aα(ω)=−1πImGαα(ω+i0+)=−1πIm〈α|G(ω+i0+)|α〉,

$G_{\alpha \alpha }$ being the *retarded* Green’s function associated to the state α defined in Equation (A2). In the absence of the scatterer, $G_{kk}\left(\omega +i0^{+}\right)=G_{0}\left(\omega +i0^{+}\right)={\left(\omega +i0^{+}-\varepsilon_{k}\right)}^{-1}$ and Equation (A10) reduces to a sum over the Fermi function $\sum_{k}f\left(\varepsilon_{k}\right)$ giving the total number of electrons $\langle N\rangle$ in the system. If the chemical potential is fixed, the introduction of the scatterer modifies the total average number of electrons. The difference with the initial one gives the amount of electrons $\langle N\rangle$ displaced by the scatterer. In the case of a single channel, one obtains:

(A12) Nwithscatterer−Nwithoutscatterer=N=−1πIm∫−∞∞dωTr[G(ω)−G0(ω)]f(ω).

Using Equation (A4) and the fact that $\frac{d}{d\varepsilon }\mathrm{Tr}\log\left[A\left(\varepsilon \right)\right]=\mathrm{Tr}\left[A^{-1}\left(\varepsilon \right)\frac{d}{d\varepsilon }A\left(\varepsilon \right)\right]$, one finds:

(A13) $$\begin{array}{cc} \langle N\rangle & =-\frac{i}{2\pi }\int_{-\infty }^{\infty }d\omega \;f\left(\omega \right)\frac{d}{d\omega }\log\det\left[I-2\pi i\nu_{0}\;T\left(\omega +i0^{+}\right)\right]\\ \; & =-\frac{i}{2\pi }\int_{-\infty }^{\infty }d\omega \;f\left(\omega \right)\frac{d}{d\omega }\log\det\;S\left(\omega +i0^{+}\right)\;, \end{array}$$

in which we applied the definition (A8) of the S-matrix. This is the general form of the Friedel sum rule. For $S\left(\varepsilon \right)=e^{2i\delta_{\varepsilon }}$ and zero temperature it gives a direct relation between the charge displaced by the impurity and the phase-shift at the Fermi energy $E_{F}$:

(A14) $$\langle N\rangle =\frac{\delta_{E_{F}}}{\pi }\;.$$

The extension to *M* channels requires to add an overall sum over the channel label σ and leads to Equation (10) in the main text.

### Appendix A.3. Illustration on the Resonant Level Model

We illustrate now the above concepts on a simple situation, sketched in Figure A1, in which the scatterer is a single resonant level tunnel coupled to chiral electrons propagating on an edge state. Such a situation is an effective representation of the mesoscopic capacitor, see Figure 7 and Figure A2, and it is described by the Coulomb Blockade Model (CBM) (12), that we remind here to the reader:

(A15) $$H_{\mathrm{CBM}}=\sum_{k}\varepsilon_{k}c_{k}^{†}c_{k}+t\sum_{k,l}\left[c_{k}^{†}d_{l}+d_{l}^{†}c_{k}\right]+\sum_{l}\left(\varepsilon_{d}+\varepsilon_{l}\right)d_{l}^{†}d_{l}+E_{c}{\left(N-N_{g}\right)}^{2}\;.$$

In this section, we neglect the last term, corresponding to interactions, and, for simplicity, we retain only a single fermionic level (annihilated by the fermion operator *d*) for the cavity, with $\varepsilon_{l}=0$. One thus obtains the Hamiltonian of a resonant level:

(A16) $$H_{\mathrm{Res}}=\sum_{k}\varepsilon_{k}c_{k}^{†}c_{k}+t\sum_{k}\left(c_{k}^{†}d+d^{†}c_{k}\right)+\varepsilon_{d}d^{†}d\;.$$

We first calculate the occupation of the cavity by calculating the retarded Green’s functions, defined as $G_{dk}\left(t-t^{\prime }\right)=-i\theta \left(t-t^{\prime }\right)\langle \left\{d\left(t\right),c_{k}^{†}\left(t^{\prime }\right)\right\}\rangle$. They are derived by solving the equations of motion in frequency space:

(A17) $$\begin{array}{cccc} \left(\omega -\varepsilon_{d}\right)G_{dd}\left(\omega \right) & =1+t\sum_{k}G_{kd}\left(\omega \right)\;, & \left(\omega -\varepsilon_{k}\right)G_{kk^{\prime }}\left(\omega \right) & =\delta_{kk^{\prime }}+tG_{dk^{\prime }}\left(\omega \right)\;, \end{array}$$

(A18) $$\begin{array}{cccc} \left(\omega -\varepsilon_{k}\right)G_{kd}\left(\omega \right) & =tG_{dd}\left(\omega \right)\;, & \left(\omega -\varepsilon_{d}\right)G_{dk}\; & =t\sum_{k^{\prime }}G_{k^{\prime }k}\left(\omega \right)\;. \end{array}$$

Solving the system, the Green’s function for the lead electrons reads:

(A19) $$G_{kk^{\prime }}\left(\omega \right)=\frac{\delta_{kk^{\prime }}}{\omega -\varepsilon_{k}}+\frac{1}{\omega -\varepsilon_{k}}t^{2}G_{dd}\left(\omega \right)\frac{1}{\omega -\varepsilon_{k^{\prime }}}\;.$$

Writing Equation (A19) in the form $G=G^{0}+G^{0}TG^{0}$, the T-matrix is found to be:

(A20) $$\begin{array}{cccc} T(\omega +i0^{+}) & =t^{2}G_{dd}\left(\omega +i0^{+}\right)=\frac{t^{2}}{\sqrt{{\left(\omega -\varepsilon_{d}\right)}^{2}+\Gamma^{2}}}e^{i\delta_{\omega }}\;, & \delta_{\omega } & =\frac{\pi }{2}-\arctan\left(\frac{\varepsilon_{d}-\omega }{\Gamma }\right)\; \end{array}$$

in which we introduced the hybridization constant:

(A21) $$\Gamma =t^{2}\sum_{k}{\left(\omega +i0^{+}-\varepsilon_{k}\right)}^{-1}∼\pi \nu_{0}t^{2}\;,$$

which corresponds to the width acquired by the resonant level by coupling to the lead and which depends on the density of states of the lead electrons at the Fermi energy $\nu_{0}$.

We can now determine the number of displaced charges 〈N〉 as given by Equation (A22) and show the validity of the Friedel sum rule (A14) in this example. In the wide-band limit, the contribution from the second term in Equation (A19) can be neglected. The number of displaced electrons is given solely by the quantum dot Green’s function $G_{dd}$:

(A22) Nwithdot−Nwithoutdot=N=−1πIm∫−∞∞dωGdd(ω)f(ω)=12−1πarctanεdΓ,

which is consistent with the Friedel sum rule (A14) with the phase-shift (A20). Equation (A22) is also meaningful because: i) It shows that, in the wide-band approximation, the number of displaced electrons $\langle N\rangle$ is given by the local Green’s function $G_{dd}$, which can be interpreted as the charge occupation of the quantum dot and ii) the number of displaced electrons depends on the orbital energy $\varepsilon_{d}$. As a consequence, a time-dependent variation of $\varepsilon_{d}$ drives a current in the system by displacing electrons in the leads.

As an additional illustration, clarifying how the scattering phase-shift appears on single particle wave-functions, we also solve explicitly the same problem in its real-space formulation. We consider chiral fermions Ψ(x) which tunnel on the resonant level of energy $\varepsilon_{d}$ (and wave-function amplitude $\Psi_{d}$), from a region of size 2η centered around x=0, useful to properly regularize the calculation and to be sent to zero at the end [240]. For lead electrons at the Fermi energy, that we set to zero (ω=0 in Equation (A20)), the Schrödinger equation reads (for x∈[−η,η]):

(A23) $$\begin{array}{cccc} 0 & =-i\hbar v_{F}\partial_{x}\Psi \left(x\right)+\frac{t}{2\eta }\Psi_{d}\;, & 0 & =\varepsilon_{d}\Psi_{d}+\frac{t}{2\eta }\int_{-\eta }^{\eta }dx^{\prime }\Psi \left(x^{\prime }\right)\;. \end{array}$$

By integrating the first equation in the interval [−η,x<η] and inserting the result for Ψ(x) in the second one, one finds:

(A24) $$\begin{array}{cccc} \Psi (x) & =\Psi \left(-\eta \right)+\frac{t}{2\eta i\hbar v}\left(x+\eta \right)\Psi_{d}\;, & \Psi_{d} & =\frac{-t}{\varepsilon_{d}-i\Gamma }\Psi \left(-\eta \right)\;. \end{array}$$

Notice that η does not appear on the last equality and can be sent safely to zero. We consider, as boundary condition for Ψ(x), incoming scattering states of the form $\Psi \left(x<0\right)=e^{ikx}/\sqrt{2\pi \hbar v_{F}}=\sqrt{\nu_{0}}e^{ikx}$. One thus finds:

(A25) $$\begin{array}{cccccc} |\Psi_{d}{|}^{2} & =\frac{1}{\pi }\frac{\Gamma }{\varepsilon_{d}^{2}+\Gamma^{2}}\;, & \Psi (0^{+}) & =\sqrt{\nu_{0}}e^{2i\delta_{0}}\;, & \delta_{0} & =\frac{\pi }{2}-\arctan\left(\frac{\varepsilon_{d}}{\Gamma }\right)\;, \end{array}$$

in agreement with Equation (A20). This short calculation illustrates how, just after scattering with the resonant level, the electron wave-packet at the Fermi energy acquires a phase $e^{2i\delta_{0}}$ which is fixed by the resonant level occupation via the Friedel sum rule. Notice that, at the resonance condition for the orbital energy ($\varepsilon_{d}=0$), $\delta_{0}=\pi /2$ and $\Psi \left(0^{+}\right)+\Psi \left(0^{-}\right)=0$, as for the unitary limit in the Kondo model, in which $\delta_{K}=\pi /2$, see also the discussion in Section 3.3.2.

### Appendix A.4. T-Matrix in the Potential Scattering Hamiltonian

We derive now the phase-shift caused by the potential scattering term on lead electrons in a local Fermi liquid (LFL). It is useful to recall here the LFL Hamiltonian (8):

(A26) $$H_{\mathrm{LFL}}=\sum_{k\sigma }\varepsilon_{k}c_{k\sigma }^{†}c_{k\sigma }+W\left(\varepsilon_{d},E_{c},\ldots \right)\sum_{k\neq k^{\prime }\sigma }c_{k\sigma }^{†}c_{k^{\prime }\sigma }\;.$$

We focus on the the single-channel case for simplicity (M=1). The generalization to σ=1,…M channels is straightforward. The Hamiltonian (A26) is quadratic and the Green’s function of the lead electrons can be readily obtained relying on the path integral formalism [79]. The partition function corresponding to Equation (A26) reads:

(A27) $$Z=\int D\left[c,c^{†}\right]e^{-S_{\mathrm{LFL}}\left[c,c^{†}\right]}\;,$$

where $S_{\mathrm{LFL}}\left[c,c^{†}\right]$ is the action of the system, which reads:

(A28) $$S_{\mathrm{LFL}}=\int_{0}^{\beta }d\tau \left\{-\sum_{k}c_{k}^{†}\left(\tau \right)G_{k}^{-1}\left(\tau \right)c_{k}\left(\tau \right)+W\sum_{k\neq k^{\prime }}c_{k}^{†}\left(\tau \right)c_{k^{\prime }}\left(\tau \right)\right\}\;,$$

where we introduced the free propagator $G_{k}^{-1}\left(\tau \right)=-\partial_{\tau }-\varepsilon_{k}$ and in which $c_{k}$ is a Grassmann variable. It is practical to switch to the frequency representation $c_{k\sigma }\left(\tau \right)=\frac{1}{\beta }\sum_{i\omega_{n}}e^{-i\omega_{n}\tau }c_{k\sigma }\left(i\omega_{n}\right)$, where we defined the fermionic Matsubara frequencies $i\omega_{n}=\left(2n+1\right)\pi /\beta$, n∈Z. They satisfy the anti-periodicity property c(β)=−c(0) and lead to:

(A29) $$S_{\mathrm{LFL}}=\sum_{i\omega_{n}}\left\{-\sum_{k}c_{k}^{†}\left(i\omega_{n}\right)G_{k}^{-1}\left(i\omega_{n}\right)c_{k}\left(i\omega_{n}\right)+W\sum_{k\neq k^{\prime }}c_{k}^{†}\left(i\omega_{n}\right)c_{k^{\prime }}\left(i\omega_{n}\right)\right\}\;,$$

with $G_{k}^{-1}\left(i\omega_{n}\right)=i\omega_{n}-\varepsilon_{k}$, which recovers the usual retarded/advanced Green’s functions by performing the analytical continuation $i\omega_{n}\to \omega \pm i0^{+}$. The full Green’s function $G_{kk^{\prime }}\left(i\omega_{n}\right)=-\langle c_{k}\left(i\omega_{n}\right)c_{k}^{†}\left(i\omega_{n}\right)\rangle$ is derived by expanding the partition function (A27) in the coupling *W* and by applying Wick’s theorem [83]. The pertubation expansion of $G_{kk^{\prime }}\left(i\omega_{n}\right)$ has the simple form:

(A30) $$\begin{array}{cc} G_{kk^{\prime }}\left(i\omega_{n}\right) & =\frac{\delta_{kk^{\prime }}}{i\omega_{n}-\epsilon_{k}}+\frac{1}{i\omega_{n}-\epsilon_{k}}\frac{1}{i\omega_{n}-\epsilon_{k^{\prime }}}W\left[1+\Sigma \left(i\omega_{n}\right)+\Sigma^{2}\left(i\omega_{n}\right)+\Sigma^{3}\left(i\omega_{n}\right)+\ldots \right]\;, \end{array}$$

in which we introduced the self-energy:

(A31) $$\begin{array}{cc} \Sigma (i\omega_{n}) & =\sum_{p}\frac{W(\varepsilon_{d})}{i\omega_{n}-\epsilon_{p}}. \end{array}$$

Using the definition (A2), the T-matrix thus reads:

(A32) $$T\left(z\right)=\frac{W}{1-\Sigma (z)}.$$

Making the analytical continuation $i\omega_{n}\to \omega +i0^{+}$ and considering a constant density of states $\nu_{0}$ for the lead electrons, we obtain:

(A33) $$\begin{array}{cc} T(\omega +i0^{+}) & =\frac{W(\varepsilon_{d})}{1+i\pi \nu_{0}W}=\frac{W(\varepsilon_{d})}{\sqrt{1+{\left[\pi \nu_{0}W\left(\varepsilon_{d}\right)\right]}^{2}}}e^{i\delta_{W}}\;, \end{array}$$

in which we introduced the phase-shift:

(A34) $$\delta_{W}=-\arctan\left(\pi \nu_{0}W\right)\;.$$

Applying the definition of the phase-shift given in Equation (A5), one finds Equation (9) in the main text. As a consistency check, substituting Equation (A33) in Equation (A8), we find that the scattering matrix reads $S=e^{2i\delta }$.

## Appendix B. Self-Consistent Description- of a 2DEG Quantum RC Circuit

In Appendix B.1, we shortly review the self-consistent scattering theory of the mesoscopic capacitor and, in Appendix B.2, we show how equivalent results can be derived with a Hamiltonian formulation.

### Appendix B.1. Self-Consistent Theory

In this section, we shortly review the description of the mesoscopic capacitor in the seminal works by Büttiker, Thomas, and Prêtre [21,51,52,53], based on a self-consistent extension of the Landauer–Büttiker scattering formalism [45,46,47]. The following discussion is also inspired from Refs. [20,241,242].

Figure A2 illustrates the intuition behind the interpretation of the mesoscopic capacitor as a quantum analog of a classical *RC* circuit. The top metallic gate (a classical metal) and the quantum dot cannot exchange electrons. These two components make up the two plates of a capacitor on which electrons accumulate according to variations of the gate potential $V_{g}$. The value of the capacitance depends on the geometry of the contact and, in the experiment of Ref. [20], the *geometrical capacitance* $C_{g}$ was estimated ∼10–100 fF. This capacitance is in series with a quantum point contact. As mentioned in the main text, direct transport measurements [160,161] consider QPCs as resistive elements of resistance $R_{\mathrm{DC}}=h/e^{2}D$, where *D* is the QPC transparency. The above considerations suggest the interpretation of the device in Figure A2 as an *RC* circuit. The admittance of a classical *RC* circuit reads:

(A35) $$A\left(\omega \right)=\frac{-i\omega C}{1-i\omega RC}=-i\omega C\left(1+i\omega CR\right)+O\left(\omega^{3}\right)\;.$$

The admittance (A35) can be calculated with the scattering formalism [45,46,47], which requires to be adapted to describe the mesoscopic capacitor in Figure A2. The main problem is the “mixed” nature of the quantum *RC* device: The mesoscopic capacitor is composed of a phase-coherent part (two-dimensional electron gas + quantum dot) in contact to an incoherent top metallic gate. As these constituents do not exchange electrons, preventing a direct current, electron transport is only possible by driving the system. We focus on the case of a gate potential oscillating periodically, as Equation (35) in the main text,

(A36) $$V_{g}\left(t\right)=V_{g}+\varepsilon_{\omega }\cos\left(\omega \;t\right)\;.$$

For small oscillation amplitudes $\varepsilon_{\omega }$, the Landauer–Büttiker formalism allows to derive the circuit admittance within linear response theory. In the case of a single conduction mode, the admittance reads [51]:

(A37) $$a\left(\omega \right)=\frac{e^{2}}{h}\int d\varepsilon \mathrm{Tr}\left[1-S^{†}\left(\varepsilon \right)S\left(\varepsilon +\hbar \omega \right)\right]\cdot \frac{f(\varepsilon )-f(\varepsilon +\hbar \omega )}{\hbar \omega }\;,$$

in which f(ε) is the Fermi distribution function, in which we fix to zero the value of the Fermi energy. For one channel, the elastic scattering assumption implies that the matrix S(ε) reduces to a pure phase $S\left(\varepsilon \right)=e^{2i\delta_{\varepsilon }}$, as electrons entering the dot return to the lead with unit probability. The phase-shift $\delta_{\varepsilon }$ is related to the dot electron occupation, via the Friedel sum rule (A14). Additionally, this phase is also related to the *dwell-time* that electrons spend in the quantum dot, or Wigner–Smith delay time [89,243]:

(A38) $$\frac{\tau (\varepsilon )}{h}=\frac{1}{2\pi i}S^{†}\left(\varepsilon \right)\frac{dS(\varepsilon )}{d\varepsilon }=\frac{1}{\pi }\frac{d\delta_{\varepsilon }}{d\varepsilon }\;.$$

The interpretation of τ as a dwell-time is illustrated in Section 4.2. In the limits T→0 and ℏω→0, Equation (A37) becomes:

(A39) $$a\left(\omega \right)=-i\omega \frac{e^{2}}{h}\left[\tau +\frac{1}{2}i\omega \tau^{2}+O\left(\omega^{2}\right)\right]\;.$$

The dwell-time τ=τ(0) is considered at the Fermi energy. Notice that this expression has the same frequency expansion as Equation (A35). Matching term by term, one finds:

(A40) $$\begin{array}{cccc} C_{q} & =\frac{e^{2}}{h}\tau \;, & R_{q} & =\frac{h}{2e^{2}}\;. \end{array}$$

Such relation between time-delays and circuit elements comes from the fact that electrons arriving on the dot at different times are differently phase-shifted, because of the variations in time of the gate potential $V_{g}$. This effect causes a local accumulation of charges, which is responsible for the emergence of quantum capacitive effects, corresponding to $C_{q}$. The time delay of the electron phase δ with respect to the driving potential U(t) is responsible for energy dissipation, controlled by $R_{q}$. The characteristic time that an electron spends in the quantum dot is given by $\tau =2R_{q}C_{q}$, twice the *RC* time because it includes the charging and relaxation time of the *RC* circuit. Notice the emergence of a universally quantized relaxation resistance, regardless of any microscopic detail of the quantum *RC* circuit, in contrast with the resistance $R_{\mathrm{DC}}=h/e^{2}D$, sensitive to the transparency *D* of the QPC and which would be measured in a DC experiment, see also Equation (43) in the main text.

Notice that the geometrical capacitance $C_{g}$ does not appear in the previous discussion. The admittance Equation (A37) has been derived by applying linear response theory for the driving potential U(t) in the quantum dot, see Figure A2. The potential U(t) does not coincide with the actual driving gate-potential $V_{g}\left(t\right)$. The situation is pictured in Figure A2: The geometric capacitance $C_{g}$ leads to a potential drop between gate and dot. In a mean-field/Hartree–Fock treatment, the potential U(t) on the dot is assumed to be uniform for each electron. This assumption is equivalent to a Random Phase Approximation (RPA), valid for weak interactions or to leading order in a 1/M expansion, with *M* the number of channels connected to the dot [78]. The potential *U* can then be determined self-consistently from the constraint of charge/current conservation in the whole device. The current $I_{\mathrm{dev}}$ flowing in the coherent part of the device has to equal the current $I_{\mathrm{gate}}$ flowing in the incoherent metallic gate:

(A41) $$I=I_{\mathrm{dev}}=I_{\mathrm{gate}}\;.$$

As all potentials are defined with respect to an arbitrary energy, they can be shifted by −eU(t), setting the potential to zero in the quantum dot. Thus, the currents in the device and in the metallic gate read:

(A42) $$\begin{array}{cccc} I_{\mathrm{dev}} & =-U(\omega )g(\omega )\;, & I_{\mathrm{gate}} & =-iC_{g}\omega \left[U\left(\omega \right)-V_{g}\left(\omega \right)\right]\;. \end{array}$$

Applying the current conservation condition (A41), the potential *U* can be eliminated, and the admittance of the total device is derived:

(A43) $$A\left(\omega \right)=-\frac{I}{V_{g}}=\frac{1}{\frac{1}{a(\omega )}+\frac{1}{-i\omega C_{g}}}\;.$$

Recalling the low frequency behavior of a(ω) in Equation (A39), the above expression shows that the whole device behaves as a RC circuit. Albeit with two capacitances in series, Equation (A43) still gives a universally quantized $R_{q}=h/2e^{2}$. The series of $C_{q}$ and $C_{g}$ gives the total capacitance *C*, originally denoted as *electro-chemical capacitance* [21,51,52,53].

We can consider a simple model for the mesoscopic capacitor to estimate the behavior of the dwell-times τ setting the quantum capacitance (A40). We consider the case of Figure A2, in which electrons propagate in the integer quantum Hall edges inside the quantum dot, see also Figure 7 in the main text. We label *ℓ* the length of the edge state in the quantum dot and $v_{F}$ the Fermi velocity of the electron. The dwell-time for electrons of velocity $v_{F}$ inside the dot is $\tau_{f}=\ell /v_{F}$. An electric wave of energy ε acquires a phase $\phi \left(\varepsilon \right)=\left(\varepsilon -eU\right)\tau_{f}/\hbar$, when making a tour of the dot. Notice that we had to shift the energy ε of the electron by −eU because of the potential shift schematized in Figure A2. The chiral nature of the edge states allows for a one-dimensional representation of the problem, pictured in the right-top of Figure 7. For a quantum well of size ℓ/2, close to the Fermi energy, the level spacing $\Delta =hv_{F}/\ell$ is constant. Thus $\tau_{f}=h/\Delta$ and, substituting in Equation (A40), leads to the uniform quantum capacitance $C_{q}=e^{2}\tau_{f}/h$, derived heuristically in the main text, see Equation (41). If the reflection amplitude at the entrance of the dot is *r* and $D=1-{\left|r\right|}^{2}$ the transmission probability, the dot can be viewed as a Fabry–Perot cavity and the phase of the out-coming electron is:

(A44) $$S\left(\varepsilon \right)=r-De^{i\phi (\varepsilon )}\sum_{q=0}^{\infty }r^{q}e^{iq\phi (\varepsilon )}=\frac{r-e^{i\phi (\varepsilon )}}{1-re^{i\phi (\varepsilon )}}=e^{i2\delta_{\varepsilon }}\;.$$

Applying Equation (A38) we obtain the local density of states:

(A45) $$N\left(\varepsilon \right)=\frac{\tau_{f}}{h}\frac{1-r^{2}}{1-2r\cos\left[\frac{2\pi }{h}\left(\varepsilon -eU\right)\tau_{f}\right]+r^{2}}\;.$$

This quantity is plotted in Figure A3 and reproduces the oscillatory behavior of the capacitance in Figure 8. In the limit of small transmission (D≪1 and r≈1), Equation (A45) reduces to a sum of Lorentzian peaks of width ℏγ, $\gamma =D/\tau_{f}$:

(A46) $$N\left(\varepsilon \right)=\frac{2}{\pi \hbar \gamma }\sum_{n}\frac{1}{1+{\left(\frac{\varepsilon -eU-n\Delta }{\hbar \gamma /2}\right)}^{2}}\;.$$

These peaks are the discrete spectrum of the dot energy levels.

The above arguments can be readily generalized to the case with *M* channels in the lead and in the cavity. In this case, every single channel σ can be considered independently. The admittance (A39) can be then cast in the form:

(A47) $$a\left(\omega \right)=-i\omega \frac{e^{2}}{h}\sum_{\sigma =1}^{M}\left[\tau_{\sigma }+\frac{1}{2}i\omega \tau_{\sigma }^{2}+O\left(\omega^{2}\right)\right]\;.$$

The low-frequency expansion of the RC circuit admittance (A35) is then recovered by defining:

(A48) $$\begin{array}{cccc} C_{q} & =\frac{e^{2}}{h}\sum_{\sigma =1}^{M}\tau_{\sigma }\;, & R_{q} & =\frac{h}{2e^{2}}\frac{\sum_{\sigma }\tau_{\sigma }^{2}}{{\left(\sum_{\sigma }\tau_{\sigma }\right)}^{2}}\;, \end{array}$$

in which $\tau_{\sigma }$ are the dwell-times in the quantum dot of electrons in the σ-th mode.

### Appendix B.2. Hamiltonian Description of the Quantum RC Circuit with a Resonant Level Model

In this appendix, we study the resonant level model (A16) as a RC circuit. The generalization of the following calculations to the many-channel case is straightforward and, in particular, we extend to the multi-level case. We thus recover the self-consistent scattering theory analysis discussed in Appendix B.1, corresponding to the limit $C_{g}\to \infty$ (non-interacting limit). Our aim is the calculation of the dynamical charge susceptibility:

(A49) $$\chi_{c}\left(t-t^{\prime }\right)=\frac{i}{\hbar }\theta \left(t-t^{\prime }\right){\langle \left[N\left(t\right),N\left(t^{\prime }\right)\right]\rangle }_{0}\;,$$

which leads to the admittance of the circuit $A\left(\omega \right)=-i\omega e^{2}\chi_{c}\left(\omega \right)$, see also Equation (36) in the main text. We make use of the path integral formalism as in Appendix A.4. The partition function corresponding to Equation (A16) reads:

(A50) $$Z=\int D\left[c,c^{†},d,d^{†}\right]e^{-S_{\mathrm{Res}}\left[c,c^{†},d,d^{†}\right]}\;,$$

where $S\left[c,c^{†},d,d^{†}\right]$ is the action of the system, which reads

(A51) $$S_{\mathrm{Res}}=\int_{0}^{\beta }d\tau \left\{-\sum_{k}c_{k}^{†}\left(\tau \right)G_{k}^{-1}\left(\tau \right)c_{k}\left(\tau \right)-d^{†}\left(\tau \right)D^{-1}\left(\tau \right)d\left(\tau \right)+t\sum_{k}\left[c_{k}^{†}\left(\tau \right)d\left(\tau \right)+c.c.\right]\right\}\;,$$

with the free propagators:

(A52) $$\begin{array}{cccc} G_{k}^{-1}\left(\tau \right) & =-\partial_{\tau }-\varepsilon_{k}\;, & D^{-1}\left(\tau \right) & =-\partial_{\tau }-\varepsilon_{d}\;, \end{array}$$

in which $c_{k}$ and $d_{l}$ are Grassmann variables. It is practical to switch to the Matsubara frequency representation, which leads to:

(A53) $$\begin{array}{c} S_{\mathrm{Res}}=\sum_{i\omega_{n}}\left\{-\sum_{k}c_{k}^{†}\left(i\omega_{n}\right)G_{k}^{-1}\left(i\omega_{n}\right)c_{k}\left(i\omega_{n}\right)-d^{†}\left(i\omega_{n}\right)D^{-1}\left(i\omega_{n}\right)d\left(i\omega_{n}\right)\right.\\ \;\;\;\left.+t\sum_{k}\left[c_{k}^{†}\left(i\omega_{n}\right)d\left(i\omega_{n}\right)+d^{†}\left(i\omega_{n}\right)c_{k}\left(i\omega_{n}\right)\right]\right\}\;, \end{array}$$

with $G_{k}^{-1}\left(i\omega_{n}\right)=i\omega_{n}-\varepsilon_{k}$ and $D^{-1}\left(i\omega_{n}\right)=i\omega_{n}-\varepsilon_{d}$. Reestablishing dimensions, the Fourier transform of Equation (A49) reads:

(A54) $$\chi_{c}\left(i\nu_{n}\right)=\frac{1}{\hbar }\int_{0}^{\hbar \beta }d\left(\tau -\tau^{\prime }\right)e^{i\nu_{n}\left(\tau -\tau^{\prime }\right)}{\langle N\left(\tau \right)N\left(\tau^{\prime }\right)\rangle }_{0}\;,$$

and $\chi_{c}\left(\omega \right)$ is recovered by performing the analytical continuation $i\nu \to \omega +i0^{+}$. This function is periodic in imaginary time and its Fourier transform is a function of the bosonic Matsubara frequencies $i\nu_{n}=2n\pi /\beta$. $N\left(\tau \right)=d^{†}\left(\tau \right)d\left(\tau \right)$ counts the number of charges on the dot. The cyclic invariance property of the trace implies that ${\langle N\left(\tau \right)N\left(\tau^{\prime }\right)\rangle }_{0}=f\left(\tau -\tau^{\prime }\right)$, allowing us to recast (A54) in the form:

(A55) $$\chi_{c}\left(i\nu_{n}\right)=\frac{1}{\beta }\sum_{i\omega_{1,2}}\langle d^{†}\left(i\omega_{1}\right)d\left(i\omega_{1}+i\nu_{n}\right)d^{†}\left(i\omega_{2}\right)d\left(i\omega_{2}-i\nu_{n}\right)\rangle \;.$$

To calculate this expression, we first perform the Gaussian integral of the lead modes in Equation (A53), leading to the effective action $S_{\mathrm{Res}}^{\prime }$ of the resonant level model:

(A56) $$\begin{array}{cccc} S_{\mathrm{Res}}^{\prime } & =-\sum_{i\omega_{n}}d^{†}\left(i\omega_{n}\right)D\left(i\omega_{n}\right)d\left(i\omega_{n}\right)\;,\; & D^{-1}\left(i\omega_{n}\right) & =i\omega_{n}-\varepsilon_{d}-t^{2}\sum_{k}G_{k}\left(i\omega_{n}\right)\;. \end{array}$$

In the wide-band approximation the propagator can be written as $D^{-1}\left(i\omega_{n}\right)=i\omega_{n}-\varepsilon_{d}+i\Gamma \mathrm{sgn}\left(\omega_{n}\right)$, where we introduced the hybridization constant $\Gamma =\pi \nu_{0}t^{2}$. The action (A56) is quadratic and the application of Wick’s theorem [83] in Equation (A55) leads to:

(A57) $$\begin{array}{cc} \chi_{c}\left(i\nu_{n}\right) & =-\frac{1}{\beta }\sum_{i\omega_{n}}D\left(i\omega_{n}\right)D\left(i\omega_{n}+i\nu_{n}\right)\to -\frac{1}{\pi \Gamma }\int_{-\infty }^{\infty }dxf\left(\Gamma x+\varepsilon_{d}\right)\frac{2x}{\left(x^{2}+1\right)\left[x^{2}-{\left(\omega /\Gamma +i\right)}^{2}\right]}\;, \end{array}$$

where the analytical continuation $i\nu \to \omega +i0^{+}$ has been performed. At zero temperature, the integral can be calculated analytically, leading to:

(A58) $$\chi_{c}\left(\omega \right)=\frac{1}{\pi \Gamma }\frac{1}{\frac{\omega }{\Gamma }\left(\frac{\omega }{\Gamma }+2i\right)}\ln\frac{\varepsilon_{d}^{2}+\Gamma^{2}}{\varepsilon_{d}^{2}-{\left(\omega +i\Gamma \right)}^{2}}\;.$$

The low frequency expansion of this expression matches the one of a classical RC circuit (A35). Reestablishing correct dimensions ω→ℏω, the result recovers Equation (A40) obtained within scattering theory:

(A59) $$\begin{array}{cccc} C_{0} & =\frac{e^{2}}{h}\nu \left(\varepsilon_{d}\right)\;, & R_{q} & =\frac{h}{2e^{2}}\;, \end{array}$$

where $\nu (\varepsilon_{d})$ is the density of states associated to the single orbital $\varepsilon_{d}$

(A60) $$\nu \left(\varepsilon_{d}\right)=\frac{1}{\pi }\frac{\Gamma }{\varepsilon_{d}^{2}+\Gamma^{2}}\;.$$

The extension to *M* channels is straightforward. One can consider a Hamiltonian of the form:

(A61) $$H_{\mathrm{Res}-\mathrm{Mch}}=\sum_{k\sigma }\varepsilon_{k}c_{k\sigma }^{†}c_{k\sigma }+t\sum_{k\sigma }\left(c_{k\sigma }^{†}d_{\sigma }+d_{\sigma }^{†}c_{k\sigma }\right)+\sum_{\sigma }\varepsilon_{\sigma }d_{\sigma }^{†}d_{\sigma }\;,$$

with σ=1,…,M the number of channels. In this model, each channel can be treated independently and one finds a a generalization of Equation (A58):

(A62) $$\chi_{c}\left(\omega \right)=\sum_{\sigma =1}^{M}\frac{1}{\pi \Gamma }\frac{1}{\frac{\omega }{\Gamma }\left(\frac{\omega }{\Gamma }+2i\right)}\ln\frac{\varepsilon_{\sigma }^{2}+\Gamma^{2}}{\varepsilon_{\sigma }^{2}-{\left(\omega +i\Gamma \right)}^{2}}\;.$$

This expression, when expanded to low frequency, has also the form (A47), where the dwell-times are substituted by the density of states of the channels (A60): $\tau_{\sigma }\to \nu_{\sigma }=\nu \left(\varepsilon_{\sigma }\right)$. One thus finds expressions for the differential capacitance and the charge relaxation resistance analog to Equation (A48):

(A63) $$\begin{array}{cccc} C_{0} & =\frac{e^{2}}{h}\sum_{\sigma =1}^{M}\nu_{\sigma }\;, & R_{q} & =\frac{h}{2e^{2}}\frac{\sum_{\sigma }\nu_{\sigma }^{2}}{{\left(\sum_{\sigma }\nu_{\sigma }\right)}^{2}}\;. \end{array}$$

#### Multi-Level Case

In this section we carry out the calculation of the quantum dot density of states in the case of a single channel and an infinite number of equally spaced levels in the quantum dot. The action reads:

(A64) $$S=\sum_{i\omega_{n}}\left\{-\sum_{k}c_{k}^{†}G_{k}^{-1}\left(i\omega_{n}\right)c_{k}-\sum_{l}d_{l}^{†}D_{l}^{-1}\left(i\omega_{n}\right)d_{l}+t\sum_{kl}\left[c_{k}^{†}d_{l}+d_{l}^{†}c_{k}\right]\right\}\;,$$

with $G_{k}^{-1}\left(i\omega_{n}\right)=i\omega_{n}-\varepsilon_{k}$ and $D_{l}^{-1}\left(i\omega_{n}\right)=i\omega_{n}-\varepsilon_{l}$. The Gaussian integration of the lead electron modes leads to the effective action:

(A65) $$S^{\prime }=\sum_{i\omega_{n}}\left\{-\sum_{l}d_{l}^{†}\left(i\omega_{n}\right)D_{l}^{-1}\left(i\omega_{n}\right)d_{l}\left(i\omega_{n}\right)+t^{2}\sum_{k}G_{k}\left(i\omega_{n}\right)\sum_{ll^{\prime }}d_{l}^{†}\left(i\omega_{n}\right)d_{l^{\prime }}\left(i\omega_{n}\right)\right\}\;.$$

Applying Wick’s theorem [83] the full propagator of the dot electrons is readily obtained:

(A66) $$D_{ll^{\prime }}\left(i\omega_{n}\right)=\delta_{ll^{\prime }}D_{l}\left(i\omega_{n}\right)+D_{l}\left(i\omega_{n}\right)D_{l^{\prime }}\left(i\omega_{n}\right)\frac{\gamma (i\omega_{n})}{1-\gamma \left(i\omega_{n}\right)\Theta \left(i\omega_{n}\right)}\;,$$

where we defined:

(A67) $$\begin{array}{cccc} \gamma (i\omega_{n}) & =t^{2}\sum_{k}G_{k}\left(i\omega_{n}\right)\;, & \Theta (i\omega_{n}) & =\sum_{l}D_{l}\left(i\omega_{n}\right)\;. \end{array}$$

In the wide band limit $\gamma \left(i\omega_{n}\right)=-i\Gamma \mathrm{sgn}\left(i\omega_{n}\right)$. The charge $\langle Q\rangle =e\sum_{l}\langle d_{l}^{†}d_{l}\rangle$ on the dot is given by:

(A68) $$\begin{array}{c} \langle Q\rangle =\frac{e}{\beta }\sum_{l,i\omega_{n}}e^{i\omega_{n}0^{+}}D_{ll}\left(i\omega_{n}\right)=\frac{e}{2\pi i}\sum_{l}\int_{-\infty }^{\infty }d\varepsilon f\left(\varepsilon \right)\left[D_{ll}\left(\varepsilon +i0^{+}\right)-D_{ll}\left(\varepsilon -i0^{+}\right)\right]\;. \end{array}$$

We write the energy spectrum on the dot as $\varepsilon_{l}=-eV_{g}+l\Delta$, with l∈Z and Δ the level spacing. Equation (A66) is then a function of $\varepsilon +eV_{g}$. Shifting all energies by $eV_{g}$, the differential capacitance $C_{0}=-\partial \langle Q\rangle /\partial V_{g}$ is readily obtained at zero temperature:

(A69) $$C_{0}=\frac{e^{2}}{2\pi i}\sum_{l}\left[D_{ll}\left(eV_{g}+i0^{+}\right)-D_{ll}\left(eV_{g}-i0^{+}\right)\right]\;,$$

with

(A70) $$\begin{array}{cc} D_{ll}\left(eV_{g}\pm i0^{+}\right) & =\frac{1}{eV_{g}-l\Delta }\mp \frac{1}{{\left(eV_{g}-l\Delta \right)}^{2}}\frac{i\Gamma }{1\pm i\Gamma \left[\sum_{p}\frac{1}{eV_{g}-p\Delta }\right]}\\ \; & =\frac{1}{\Delta }\left\{\frac{1}{x+l}\mp \frac{i\Gamma /\Delta }{{\left(x+l\right)}^{2}}\frac{1}{1\pm i\pi \frac{\Gamma }{\Delta }\coth\left(\pi x\right)}\right\}\;, \end{array}$$

where $x=eV_{g}/\Delta$ and we exploited the fact that $\sum_{l}\frac{1}{x+l}=\Psi_{0}\left(1-x\right)-\Psi_{0}\left(x\right)=\pi \coth\left(\pi x\right)$, in which $\Psi_{0}\left(x\right)$ is the digamma function. Substituting this expression in Equation (A69), the sum over levels can be also carried out, leading to:

(A71) $$C_{0}=e^{2}\frac{\pi \Gamma }{2\Delta^{2}}\frac{1}{\sin^{2}\left(\pi \frac{eV_{g}}{\Delta }\right)}\left[\frac{1}{1+i\pi \frac{\Gamma }{\Delta }\coth\left(\pi \frac{eV_{g}}{\Delta }\right)}+\frac{1}{1-i\pi \frac{\Gamma }{\Delta }\coth\left(\pi \frac{eV_{g}}{\Delta }\right)}\right]\;,$$

where we relied on the identity: $\sum_{l}\frac{1}{{\left(l+x\right)}^{2}}=\Psi_{1}\left(1-x\right)-\Psi_{1}\left(x\right)=\frac{\pi^{2}}{\sin^{2}\left(\pi x\right)}$, in which $\Psi_{n}\left(x\right)$ is the polygamma function. Some algebra leads to:

(A72) $$C_{0}=\frac{e^{2}}{\Delta }\frac{2}{\frac{\Delta }{\pi \Gamma }+\frac{\pi \Gamma }{\Delta }-\left(\frac{\Delta }{\pi \Gamma }-\frac{\pi \Gamma }{\Delta }\right)\cos\left(\frac{2\pi eV_{g}}{\Delta }\right)}\;.$$

This quantity is plotted in Figure A3 as a function of the gate potential $V_{g}$ and reproduces the oscillations of the capacitance observed in Figure 8, in the main text. As we did not consider any many-body interaction to derive $C_{0}$, this quantity corresponds to the quantum capacitance $C_{q}=e^{2}N\left(E_{F}\right)$ corresponding to the density of states at the Fermi level, see also discussion in Section 4.2. Such density of states was also derived within scattering theory in Appendix B.1. Indeed, Equations (A45) and (A72) coincide if one makes the identification (It is useful to recall here that $\tau_{f}=h/\Delta$)

(A73) $$\begin{array}{cccccc} \frac{\pi \Gamma }{\Delta } & =\frac{{\left(1-r\right)}^{2}}{1-r^{2}}=\frac{1-r}{1+r} & \Leftrightarrow & \; & r & =\frac{1-\frac{\pi \Gamma }{\Delta }}{1+\frac{\pi \Gamma }{\Delta }}\;. \end{array}$$

Notice that the fully transparent limit coincides with πΓ/Δ=1, corresponding to a change of sign of the reflection amplitude *r* (remind that we assumed *r* to be a real number). Additionally, if we consider the tunneling limit πΓ/Δ≪1, we can write $r=\sqrt{1-D}$ and, in the low-transparency limit D≪1 one recovers πΓ/Δ=D/4, which is consistent with the expectation $D\propto t^{2}$ in the tunneling limit of the Hamiltonian (A16). Notice also that the relation (A73) implies r=−1 in the Γ→∞ limit, which can be explained by the formation of bonding and anti-bonding states at the junction between electrons in the lead and in the dot, suppressing tunneling in the dot [240]. For a single level and one channel, we recover the universal charge relaxation resistance $R_{q}=h/2e^{2}$.

## Appendix C. Useful Results of Linear Response Theory

In this appendix we remind some useful properties of linear response theory following Ref. [244]. In Appendix C.1, we show that the real/imaginary parts of the dynamical charge susceptibility (A49) are respectively even/odd functions of the frequency, leading to:

(A74) A(ω)=−iωe2χc+iImχc(ω)+O(ω2),

that is Equation (38) in the main text. In Appendix C.2, we demonstrate that the power dissipated by the quantum *RC* circuit in the linear response regime is given by:

(A75) P=12εω2ωImχc(ω),

that is Equation (55) in the main text.

### Appendix C.1. Parity of the Dynamical Charge Susceptibility

The Lehman representation [97] of the dynamical charge susceptibility $\chi_{c}\left(\omega \right)$ (A49) makes explicit its real and imaginary parts. This is obtained from the Fourier transform of Equation (A49):

(A76) $$\chi_{c}\left(\omega \right)=\frac{i}{\hbar }\int_{-\infty }^{\infty }d\left(t-t^{\prime }\right)e^{i\left(\omega +i0^{+}\right)\left(t-t^{\prime }\right)}\theta \left(t-t^{\prime }\right){\langle \left[N\left(t\right),N\left(t^{\prime }\right)\right]\rangle }_{0}\;,$$

where the factor $i0^{+}$ is inserted to regularize retarded functions. Inserting the closure relation with the eigenstates $|n\rangle$ of energy $E_{n}$ of the time independent Hamiltonian $H_{0}$, the average can be written as:

(A77) $${\langle N\left(t\right)N\left(t^{\prime }\right)\rangle }_{0}=\sum_{n,m}p_{n}e^{i\omega_{nm}\left(t-t^{\prime }\right)}N_{nm}N_{mn}\;,$$

where $p_{n}=e^{-\beta E_{n}}/Z$ is the Boltzmann weight, $\hbar \omega_{nm}=E_{n}-E_{m}$ and $N_{nm}=\langle n|N|m\rangle$ the matrix elements of the dot occupation. In this representation, the Fourier transform (A76) reads:

(A78) $$\chi_{c}\left(\omega \right)=-\frac{1}{\hbar }\sum_{nm}p_{n}N_{nm}N_{mn}\left(\frac{1}{\omega +i0^{+}+\omega_{nm}}-\frac{1}{\omega +i0^{+}-\omega_{nm}}\right)\;.$$

Applying the relation $\frac{1}{x\pm i0^{+}}=P\left[\frac{1}{x}\right]\mp i\pi \delta \left(x\right),$ with P[f(x)] the principal value of the function f(x), the real and imaginary part of $\chi_{c}\left(\omega \right)$ are readily obtained:

(A79) Reχc(ω)=−1ℏ∑nmpnNnmNmnP1ω+ωnm−P1ω−ωnm,

(A80) Imχc(ω)=iπℏ∑nmpnNnmNmnδω+ωnm−δω−ωnm,

which are respectively an even and odd function of ω. As a consequence, in the low frequency expansion of the dynamical charge susceptibility $\chi_{c}\left(\omega \right)=\chi_{c}\left(0\right)+\omega \partial_{\omega }\chi_{c}{\left(\omega \right)|}_{\omega =0}+O\left(\omega^{2}\right)$, the linear term in ω has to coincide with the imaginary part of Im[χc(ω)], leading to Equation (A74).

### Appendix C.2. Energy Dissipation in the Linear Response Regime

In the situation addressed in Section 4, the time dependence of orbital energies in the dot is given by $\varepsilon_{d}\left(t\right)=\varepsilon_{d}^{0}+\varepsilon_{\omega }\;\cos\left(\omega t\right)$. In the time unit, the systems dissipates the energy:

(A81) $$\delta W=\delta \langle N\rangle \varepsilon_{\omega }\;\cos\left(\omega t\right)\;.$$

In the stationary regime, the average power P dissipated by the system during the time period *T* reads:

(A82) $$P=\frac{\varepsilon_{\omega }}{T}\int_{0}^{T}dt\;\frac{d\langle N(t)\rangle }{dt}\;\cos\left(\omega t\right)\;.$$

Neglecting constant contributions, $\langle N(t)\rangle$ is given by the dynamical charge susceptibility (A49):

(A83) $$\langle N(t)\rangle =\varepsilon_{\omega }\int_{-\infty }^{\infty }dt^{\prime }\chi_{c}\left(t-t^{\prime }\right)\cos\left(\omega t\right)\;.$$

Substituting this expression in Equation (A82), we obtain:

(A84) $$P=-i\omega \frac{\varepsilon_{\omega }^{2}}{4}\left[\chi_{c}\left(\omega \right)-\chi_{c}\left(-\omega \right)\right]+i\omega \frac{\varepsilon_{\omega }^{2}}{T}\int_{0}^{T}dt\frac{\chi_{c}\left(-\omega \right)e^{2i\omega t}-\chi_{c}\left(\omega \right)e^{-2i\omega t}}{4}\;.$$

Expressing χc(ω)=Reχc(ω)+iImχc(ω) as the sum of its real and imaginary part and applying the parity properties demonstrated in Appendix C.1, the first term recovers Equation (A75) for the dissipated power:

(A85) P=12εω2ωImχc(ω),

while the second term in Equation (A84) reduces to vanishing integrals of sin(2ωt) and cos(2ωt) over their period. In the case of Section 4.5, describing the energy dissipated by the LFL effective low-energy theory, we can apply the same considerations by replacing $\delta \langle N\rangle$ in Equation (A81) with the average of the operator *A*, defined in Equation (57). One thus derives Equation (56) in the main text.
